# Isobornylchalcones as Scaffold for the Synthesis of Diarylpyrazolines with Antioxidant Activity

**DOI:** 10.3390/molecules26123579

**Published:** 2021-06-11

**Authors:** Svetlana A. Popova, Evgenia V. Pavlova, Oksana G. Shevchenko, Irina Yu. Chukicheva, Aleksandr V. Kutchin

**Affiliations:** 1 Institute of Chemistry, Komi Science Centre, Ural Branch, Russian Academy of Sciences, 48 Pervomayskaya, 167000 Syktyvkar, Russia; evgenia.pavlova92@rambler.ru (E.V.P.); chukicheva-iy@chemi.komisc.ru (I.Y.C.); kutchin-av@mail.ru (A.V.K.); 2 Institute of Biology, Komi Science Centre, Ural Branch, Russian Academy of Sciences, 28 Kommunisticheskaya, 167982 Syktyvkar, Russia; shevchenko@ib.komisc.ru

**Keywords:** isobornylchalcones, diarylpyrazolines, Fe^2+^/ascorbate-initiated lipid peroxidation, antioxidant activity

## Abstract

The pyrazoline ring is defined as a “privileged structure” in medicinal chemistry. A variety of pharmacological properties of pyrazolines is associated with the nature and position of various substituents, which is especially evident in diarylpyrazolines. Compounds with a chalcone fragment show a wide range of biological properties as well as high reactivity which is primarily due to the presence of an α, β-unsaturated carbonyl system. At the same time, bicyclic monoterpenoids deserve special attention as a source of a key structural block or as one of the pharmacophore components of biologically active molecules. A series of new diarylpyrazoline derivatives based on isobornylchalcones with different substitutes (MeO, Hal, NO_2_, N(Me)_2_) was synthesized. Antioxidant properties of the obtained compounds were comparatively evaluated using in vitro model Fe^2+^/ascorbate-initiated lipid peroxidation in the substrate containing brain lipids of laboratory mice. It was demonstrated that the combination of the electron-donating group in the *para*-position of ring B and OH-group in the ring A in the structure of chalcone fragment provides significant antioxidant activity of synthesized diarylpyrazoline derivatives.

## 1. Introduction

Lipid peroxidation (LPO) is a process initiated by free radicals attacking phospholipids or polyunsaturated fatty acids, which leads to the formation of various types of toxic oxidation products [1]. These highly reactive products, through interaction with cellular components, can initiate the mechanisms of several disorders and diseases, such as cardiovascular, neurodegenerative diseases, cancer and aging [2]. Thus, the design and development of new antioxidants for the prevention and treatment of the above-mentioned diseases are becoming increasingly important.

**Figure 1:**
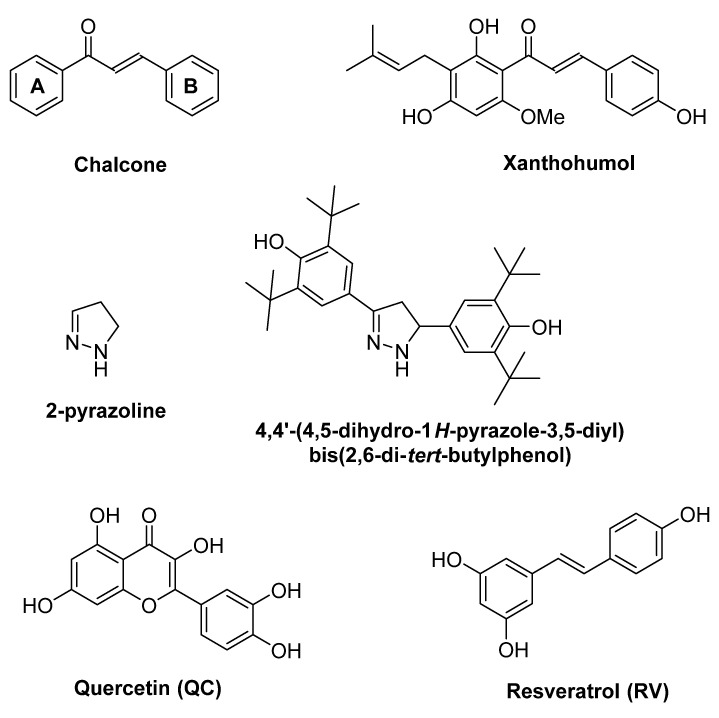
Chemical structures of some biologically active compounds.

Chalcones (1,3-diphenyl-2-propen-1-ones) are a subclass of open-chain flavonoids that are present in many plants [3]. Structurally, chalcones consist of two aromatic rings (A and B) linked by a three-carbon α-β unsaturated carbonyl moiety (Figure 1). The chalcone skeleton is considered a privileged scaffold in medicinal chemistry and is widely used as an effective template for drug discovery [4]. Compounds with a chalcone fragment exhibit various types of biological activity such as antibacterial, antifungal, anti-inflammatory, anti-cancer, etc. [5,6,7,8,9,10].

It was demonstrated that synthetic chalcone derivative 2-hydroxy-4′-methoxychalcone (AN07) potentially has anti-atherosclerosis effects, as well as antioxidant, anti-inflammatory, and neuroprotective effects [11]. Some prenylchalcones are isolated from hops and beer and exhibit antioxidant effects, modulate metabolism of carcinogens by inhibition of distinct phase 1 metabolic enzymes and activation of phase 2 detoxifying enzymes, and display anti-inflammatory properties [12]. Xanthohumol, the main prenylchalcone of hops and beer, showed a high antioxidant activity (AOA) of inhibiting the oxidation of low density lipoproteins (LDL), greater than α-tocopherol and isoflavone genistein, but less than flavonol quercetin (Figure 1) [13]. Chalcones are highly reactive due to the presence of two active electrophilic centers, the carbonyl group and the double bond conjugated to it, and can react as ambident electrophiles due to the delocalization of the electron density in the three-carbon α-β unsaturated carbonyl system. In addition, these compounds are of great interest as available starting reagents for reactions involving binucleophiles, leading to a wide variety of 5-, 6-, and 7-membered carbo- and heterocyclic compounds, such as benzodiazepines, dihydropyrimidines, pyrazolines, etc. [14,15,16,17].

Amongst these heterocyclic ring-containing scaffolds, pyrazolines as a class of electron-rich nitrogen heterocyclic compounds play an important role due to their extensive use as pharmacophore and synthon. Interestingly, 2-pyrazoline derivatives synthesized from chalcones have shown a variety of biological activities, such as antibacterial, antitumor, antifungal, anti-inflammatory, antioxidant, and antimalarial [18,19,20,21,22,23,24]. Previously it has been reported that 3-(3,5-di-*tert*-butyl-4-hydroxyphenyl)-5-(multi-substituted-4-hydroxyphenyl)-2-pyrazolines showed significant human LDL-antioxidant activities (Figure 1) [25]. These results demonstrated that bulky di-*tert*-butyl groups contribute to higher activity by creating steric and electronic factors to stabilize the phenoxy radical formed from the phenolic hydroxy group, which could affect the antioxidant activity of human low-density lipoproteins.

At the same time, the hybridization of biologically active molecules, based on the combination of pharmacophore groups of two or more known biologically active compounds, is a powerful strategy for drug development. It leads to the development of new hybrid compounds that preserve the pre-selected characteristics of the original templates. Previously, it was demonstrated that the introduction of a terpene fragment into the 4-methylcoumarin scaffold increased the antioxidant, antiradical, and membrane-protective activity of the coumarin derivatives [26]. The 3,4-dihydro-2*H*benz[*e*][1,3]oxazine derivative of 2-hydroxy-3-isobornyl-5-methylbenzaldehyde showed high membrane-protective activity on the model H_2_O_2_-induced hemolysis towards mammalian red blood cells [27]. From this point of view, the synthesis of heterocyclic compounds based on the transformations of isobornylphenols and the study of their antioxidant activity is an interesting subject of research.

Heterogeneous systems, particularly oil–water emulsions, are often used to study antioxidant activity [28,29,30,31,32,33]. The interfacial properties of derivatives affected by the type of substituting group is the predominant factor to exert antioxidant activity in this model [31,32]. A suitable and affordable source of easily oxidized lipids for preparation of model emulsions is the brain of laboratory animals. Brain homogenate is a substrate widely used as an oxidative stress model [34,35,36,37,38]. The brain is extremely vulnerable to oxidative stress, in part because it is highly enriched with non-heme iron, which is catalytically involved in the production of oxygen free radicals. In addition, the brain contains a relatively high degree of polyunsaturated fatty acids that are particularly good substrates for peroxidation reactions [39,40]. This approach is common for studies of antioxidant activity of food products, plant extracts, and chemical compounds promising for pharmacology. We regularly use this method to assess the antioxidant activity of compounds of various structures [26,27,41,42].

This work describes the synthesis of diarylpyrazoline derivatives with an isobornyl substituent and a study of their antioxidant activity using the model of Fe^2+^/ascorbate-initiated LPO in substrate obtained from mice brain homogenate. Quercetin and resveratrol were used as a standard.

## 2. Results and Discussion

**Scheme 1:**
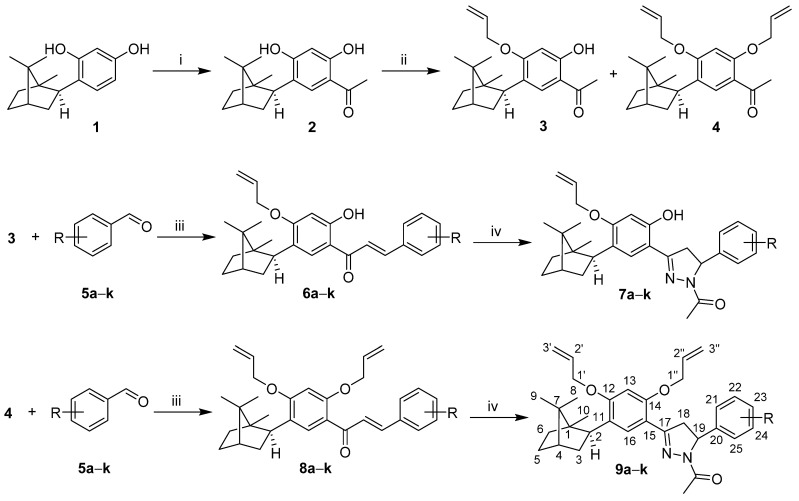
Synthesis of pyrazoline derivatives **7a**–**k** and **9a**–**k**. Reagents and conditions: (i) Ac_2_O, BF_3_·Et_2_O, 60 °C, 3 h, 76%; (ii) (CH_3_)_2_CO, AllylBr, K_2_CO_3_/KI, heat; (iii) for **6a**–**d**, **8a**–**d** 40% (*w*/*v*) sodium hydroxide, methanol, rt; for **6e**–**k**, **8e**–**k** NaH, DMF, 0–25 °C; (iv) hydrazine hydrate, CH_3_COOH, rf.

The initial racemic 1,3-dihydroxy-4-isobornylbenzene **1** was synthesized via the alkylation of resorcinol with camphene according to the known method [43] (Scheme 1). Acetophenones **3** and **4** were obtained by acetylation of compound **1** with acetic anhydride in BF_3_ Et_2_O followed by *O*-allylation of the resulting product **2** [44].

Claisen–Schmidt condensation of isobornylacetophenone derivatives **3** and **4** with appropriately substituted benzaldehydes was carried out in order to synthesize a set of chalcone derivatives with dimethylamino, chloro, bromo, methoxy, and nitro B-ring substituents (Scheme 1). The synthesis of chalcones **6a**–**k** and **8a**–**k** has been previously described [44,45]. Chalcones **6a**–**d** and **8a**–**d** were synthesized by KOH/MeOH condensation of compounds **3** and **4** with appropriate benzaldehydes, methoxychalcones **6e**–**k**, **8e**–**k** by condensation of compounds **3** and **4** with methoxy-substituted benzaldehydes **5e**–**k** in the presence of sodium hydride in dimethylformamide. The reaction of chalcones **6a**–**k**, **8a**–**k** with hydrazine in acetic acid under reflux condition produced the corresponding pyrazoline derivatives **7a**–**k**, **9a**–**k** (Scheme 1, Table 1).

The structure of new substituted diarylpyrazolines with an isobornyl moiety was established on the basis of ^1^H and ^13^C NMR spectroscopy and mass spectrometry. In the ^1^H NMR spectra of compounds **7a**–**k** and **9a**–**k**, there are no signals of the vinyl protons of the unsaturated α-β bond in the region of δH 7.41–8.39 ppm, but there is a signal of the CH_3_ group of the *N*-acyl fragment in the region of δ_H_ 2.28–2.46 ppm. Signals of the methylene (in the range of 3.08–3.33 and 3.84–3.99 ppm) and methine (5.49–5.96 ppm) groups of the pyrazoline ring are also observed. The integrated intensity of the aromatic proton signals corresponds to the declared structures. The ^13^C NMR spectra contain signals of the CH_3_ carbon atom of the N-acyl group at 21–22 ppm and signals of the methine (57 ppm) and methylene (42 ppm) groups are observed, the signal of the C=O group carbon atom is present in the weak field region of 210 ppm. Copies of ^1^H and ^13^C NMR spectra of compounds **7a,b,i,j** and **9a,b,i,k** are provided in Supplementary Materials. Mass spectral data are in accordance with the proposed structures.

**Figure 2:**
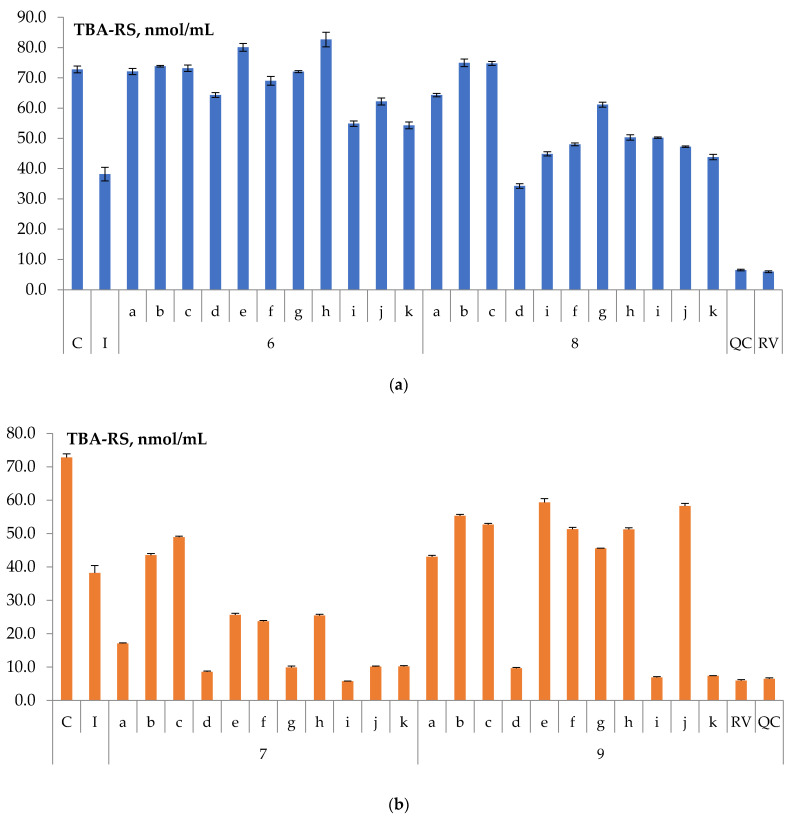
Antioxidant activity of chalcones and pyrazolines: (**a**) Chalcones **6a**–**k** and **8a**–**k**; (**b**) Pyrazolines **7a**–**k** and **9a**–**k**. C—control; I-intact; RV—resveratrol; QC—quercetin.

Figure 2 shows the results of a comparative assessment of the antioxidant activity of 44 chalcones and diarylpyrazolines. The AOA of compounds was evaluated as inhibition of accumulation of secondary LPO products (TBA-RS) in substrates. In general, pyrazoline derivatives (Figure 2b) exhibit greater activity compared to the corresponding chalcones (Figure 2a) containing an α-β-unsaturated carbonyl system, which indicates the leading role of 4,5-dihydro-1*H*-pyrazole scaffold in the manifestation of antioxidant function of the compounds under consideration. At the same time, for both chalcones and arylpyrazolinesa significant dependence of the activity on the structure, number and position of substituents in both phenyl cores (A and B) was observed.

Thus, in this model system, no AOA was detected for chalcones containing nitro group (**6a** and **8a**) or halogen atoms (**6b**,**c** and **8b**,**c**) in the aromatic ring B. The presence of donor methoxy groups in this ring promotes antioxidant activity, and not only their number but also their position is essential. In addition to methoxy groups, the antioxidant activity of chalcones can also be impacted by the dimethylamine group at the C-4 position of the B ring. Interestingly, in such a structure, high activity was detected only in chalcone **8d**, but not in chalcone **6d**, which differ in substituents in ring A. The influence of the A-ring substituents’ structure on the AOA of chalcones is also noted for the above-mentioned compounds. The presence of two allyl substituents (**8e**–**k**) had a greater effect than the combination of allyl and hydroxyl substituents (**6e**–**k**) with other matching structures.

Among the diarylpyrazolines, the compounds containing the halogen atom (**7b**,**c** and **9b**,**c**) in the B ring were also the least active. Diarylpyrazolines with a nitro group in ring B (**7a** and **9a**) turned out to be more active than the corresponding chalcones (**6a** and **8a**). As with chalcones, the antioxidant activity of diarylpyrazolines is associated with the presence of electron-donating substituent in the *para*-position of this ring. For instance, high AOA was also found in diarylpyrazolines with a dimethylamine group at position C-4 of ring B (**7d** and **9d**). Among mono methoxy pyrazolines, the most active were compounds with a substituent in the *para*-position of ring B, while the *ortho*- and *meta*-isomers showed similar results of activity. Moreover, the structure of the substituents in ring A is also important here. In all cases, compounds with a hydroxyl group in the C-2 position of ring A turned out to be more active than the corresponding derivatives with two allyloxy substituents. For diarylpyrazolines with two methoxy groups in ring B, the compounds with the catechol moiety **7i** and **9i** were predictably most active. It should be noted that these compounds are leaders in antioxidant activity among all studied compounds and showed significant ability to inhibit LPO at the level of values for standards. The patterns revealed above were also valid for diarylpyrazolines with three methoxy groups (**7j**,**k** and **9j**,**k**). In contrast to the first three compounds (**7g**,**k** and **9k**), which showed high AOA, compound **9j**, combining two allyloxy substituents in ring A and 2,4,6-trimethoxy substituents in ring B, inhibited LPO to a small extent.

The decrease in Fe^2+^-induced lipid peroxidation in substrate obtained from mouse brain homogenate in the presence of pyrazolines could be the result of their ability to chelate Fe^2+^ and/or radical scavenging activity. Compound **7i** was the most effective antioxidant. These results strongly suggest that the presence of hydroxyl group at 2′-position in ring A (compare **7a**–**k** to **9a**–**k**) and the catechol moiety in ring B (**7i** and **9i**) are essential for inhibiting Fe^2+^/ascorbate-mediated LPO in this model system.

## 3. Materials and Methods

### 3.1. Chemistry

The ^1^H- and ^13^C-NMR spectra were recorded on a Avance II 300 instrument (300 MHz and 75 MHz, (Bruker CorporationGermany) in CDCl_3_. The assignment of the atoms’ signals of synthesized compounds was carried out using the ^1^H and J-modulated ^13^C NMR spectra, as well as using the HSQC, HMBC, NOESY, COSY techniques. The melting points were measured on a Gallenkamp MPD 350 instrument (Sanyo, Moriguchi, Japan) and were not corrected. Mass spectra were recorded on a Thermo Finnigan LCQ Fleet instrument (Thermo Fisher Scientific, Waltham, MA, USA). The reaction progress was monitored by thin layer chromatography (TLC) on Sorbfil plates. Column chromatography was carried out on silica gel Alfa Aesar 70/230 μ (Alfa Aesar, Ward Hill, MA, USA).

The spectral data were partially obtained using the equipment of the Center of Collective Usage Chemistry (Institute of Chemistry, Komi Scientific Centre, Ural Branch of the RAS, Syktyvkar, Russia).

Synthesis and spectral characteristics of compounds **1**–**4, 6a**–**k,** and **8a**–**k** have been described previously [43,44].

#### General Procedure for the Synthesis of Pyrazolines

A mixture of chalcone (1 mmol), hydrazine monohydrate (5 mmol), and acetic acid (6 mL) were refluxed for 1–2.5 h. The progress of the reaction was monitored by TLC. The resulting mixture was poured into ice-cold water andallowed to stand. The precipitate that formed was separated by filtration and washed with cold water. In cases where no precipitate was formed, the mixture was extracted with ethyl acetate (3 × 10 mL). The organic extracts were dried over anhydrous sodium sulphate, filtrated, and evaporated under vacuum. Additional purification of the reaction product was carried out by column chromatography on silica gel.

*1-(5-(3-Nitrophenyl)-3-(4′-allyloxy-2′-hydroxy-5′-isobornylphenyl)-4,5-dihydro-(1H)-pyrazole-1-yl)etanone* (**7a**). Yellow oil; 78% yield. IR (KBr), ν/cm^−1^ 3415 (OH), 1668 (C=O), 1625 (C=N), 1348 (N−O) 1261 (=C−O), 1247 (C−N). ^1^H NMR (CDCl_3_, δ ppm, *J*/Hz): 0.71 (s, 3H, 10-CH_3_); 0.85 (s, 3H, 8-CH_3_); 0.86 (s, 3H, 9-CH_3_); 1.29−1.46 (m, 2H, 5-CH_2_, 6-CH_2_); 1.57−1.61 (m, 2H, 3-CH_2_, 6-CH_2_); 1.82−1.84 (m, 2H, 5-CH_2_, 4-CH); 2.06−2.11 (m, 1H, 3-CH_2_); 2.43 (s, 3H, N-COCH_3_); 3.26−3.33 (m, 2H, 18-CH_2_, 2-CH); 3.89−3.99 (m, 1H, 18-CH_2_); 4.59 (d, *J* = 5 Hz, 2H, 1′-CH_2_); 5.36 (d, *J* = 11 Hz, 1H, 3′-CH_2_(H*cis*)); 5.50−5.53 (d, *J* = 17 Hz, 1H, 3′-CH_2_(H*trans*)); 5.62−5.68 (m, 1H, 19-CH); 6.07−6.13 (m, 1H, 2′-CH); 6.57 (s, 1H, 13-CH); 7.11 (s, 1H, 16-CH); 7.53−7.64 (m, 2H, 24-CH, 25-CH); 7.48−7.51 (m, 2H, 21-CH, 23-CH); 10.17 (s, 1H, C(14)-OH). ^13^C NMR (CDCl_3_, δ ppm): 12.2 (10-CH_3_); 20.1 (8-CH_3_); 21.5 (9-CH_3_); 21.9 (N-COCH3); 27.4 (5-CH_2_); 34.1 (3-CH_2_); 39.4 (6-CH_2_); 42.7 (18-CH_2_); 44.1 (2-CH); 45.5 (4-CH); 48.1 (7-C); 49.6 (1-C); 57.6 (19-CH-N); 68.9 (1′-CH_2_); 99.9 (13-CH); 106.5 (11-C); 117.6 (3′-CH_2_); 120.9 (21-CH); 122.9 (25-CH); 124.5 (15-C); 127.5 (16-CH); 130.1 (24-CH); 131.9 (23-CH); 132.6 (2′-CH); 143.5 (20-C); 146.3 (22-C); 156.5 (17-C=N); 160.2 and 167.9 (12-C) and (14-C); 211.1 (C=O). ESI-MS *m*/*z*: found 518.71 [M + H]^+^, calcd. for C_30_H_36_N_3_O_5_ 518.62.

*1-(5-(4-Chlorophenyl)-3-(4′-allyloxy-2′-hydroxy-5′-isobornylphenyl)-4,5-dihydro-(1H)-pyrazole-1-yl)etanone* (**7b**). Yellow oil; 70% yield. IR (KBr), ν/cm^−1^ 3431 (OH), 1662 (C=O), 1627 (C=N), 1263 (=C−O), 1257 (C−N), 1022 (Ar−Cl). ^1^H NMR (CDCl_3_, δ ppm, *J*/Hz): 0.74 (s, 3H, 10-CH_3_); 0.83 (s, 3H, 8-CH_3_); 0.84 (s, 3H, 9-CH_3_); 1.29−1.39 (m, 2H, 5-CH_2_, 6-CH_2_); 1.48−1.58 (m, 2H, 3-CH_2_, 6-CH_2_); 1.61−1.83 (m, 2H, 5-CH_2_, 4-CH); 2.01−2.11 (m, 1H, 3-CH_2_); 2.39 (s, 3H, N-COCH_3_); 3.12−3.19 (m, 1H, 18-CH_2_); 3.28 (t, *J* = 9.0 Hz, 1H, 2-CH); 3.84−3.94 (m, 1H, 18-CH_2_); 4.59 (d, *J* = 4.9 Hz, 2H, 1′-CH_2_); 5.37 (d, *J* = 10.9 Hz, 1H, 3′-CH_2_(H*cis*); 5.49−5.56 (m, 2H, 3′-CH_2_(H*trans*), 19-CH); 6.07−6.13 (m, 1H, 2′-CH); 6.56 (s, 1H, 13-CH); 7.11 (s, 1H, 16-CH); 7.23 (d, *J* = 8 Hz, 2H, 21-CH, 25-CH); 7.31 (d, *J* = 8.3 Hz, 2H, 22-CH, 24-CH); 10.25 (s, 1H, C(14)-OH). ^13^C NMR (CDCl_3_, δ ppm): 12.4 (10-CH_3_); 20.3 (8-CH_3_); 21.5 (9-CH_3_); 22.1 (N-COCH_3_); 27.4 (5-CH_2_); 34.3 (3-CH_2_); 39.6 (6-CH_2_); 42.7 (18-CH_2_); 44.3 (2-CH); 45.6 (4-CH); 48.1 (7-C); 49.5 (1-C); 57.7 (19-CH-N); 68.8 (1′-CH_2_); 99.9 (13-CH); 106.9 (11-C); 117.5 (3′-CH_2_); 124.5 (15-C); 127.2 (21-CH, 25-CH); 127.6 (16-CH); 129.2 (22-CH, 24-CH); 132.1 (2′-CH); 132.6 (20-C); 139.9 (23-C); 156.6 (17-C=N); 160.1 and 167.8 (12-C) and (14-C); 211.3 (C=O). ESI-MS *m*/*z*: found 507.95 [M + H]^+^, calcd. for C_30_H_36_ClN_2_O_3_ 508.06.

*1-(5-(4-Bromophenyl)-3-(4′-allyloxy-2′-hydroxy-5′-isobornylphenyl)-4,5-dihydro-(1H)-pyrazole-1-yl)etanone* (**7c**). Gray-yellow powder; 91% yield; m.p. 73–74 °C. IR (KBr), ν/cm^−1^ 3421 (OH), 1668 (C=O), 1625 (C=N), 1261 (=C−O), 1245 (C−N). ^1^H NMR (CDCl_3_, δ ppm, *J*/Hz): 0.73 (s, 3H, 10-CH_3_,); 0.82 (s, 3H, 8-CH_3_,); 0.92 (s, 3H, 9-CH_3_,); 1.29−1.47 (m, 2H, 5-CH_2_, 6-CH_2_); 1.52−1.61 (m, 2H, 3-CH_2_, 6-CH_2_); 1.68−1.83 (m, 2H, 5-CH_2_, 4-CH); 2.02−2.06 (m, 1H, 3-CH_2_); 2.39 (s, 3H, N-COCH_3_); 3.12−3.28 (m, 2H, 18-CH_2_, 2-CH); 3.84−3.95 (m, 1H, 18-CH_2_); 4.60 (d, *J* = 4.8 Hz, 2H, 1′-CH_2_); 5.37 (d, *J* = 11 Hz, 1H, 3′-CH_2_(H*cis*)); 5.50−5.55 (m, 2H, 3′-CH_2_(H*trans*), 19-CH); 6.07−6.13 (m, 1H, 2′-CH); 6.55 (s, 1H, 13-CH); 7.11 (s, 1H, 16-CH); 7.14−7.19 (m, 2H, 21-CH, 25-CH); 7.48−7.51 (m, 2H, 22-CH, 24-CH); 10.25 (s, 1H, C(14)-OH). ^13^C NMR (CDCl_3_, δ ppm): 12.4 (10-CH_3_); 20.1 (8-CH_3_); 21.5 (9-CH_3_); 22.1 (N-COCH3); 27.4 (5-CH_2_); 34.2 (3-CH_2_); 39.6 (6-CH_2_); 42.7 (18-CH_2_); 44.2 (2-CH); 44.3 (4-CH); 45.5 (7-C); 48.8 (1-C); 57.7 (19-CH-N); 68.8 (1′-CH_2_); 99.9 (13-CH); 106.9 (11-C); 117.5 (3′-CH_2_); 124.5 (15-C); 127.5 (21-CH, 25-CH); 127.6 (16-CH); 132.1 (22-CH, 24-CH); 132.7 (2′-CH); 132.6 (20-C); 140.5 (23-C); 156.8 (17-C=N); 16.1 and 168.8 (12-C) and (14-C); 210.8 (C=O). ESI-MS *m*/*z*: found 552.35 [M + H]^+^, calcd. for C_30_H_36_BrN_2_O_3_ 552.51.

*1-(5-(4-Dimethylaminophenyl)-3-(4′-allyloxy-2′-hydroxy-5′-isobornylphenyl)-4,5-dihydro-(1H)-pyrazole-1-yl)etanone* (**7d**). Yellow oil; 71% yield. IR (KBr), ν/cm^−1^ 3396 (OH), 1664 (C=O), 1624 (C=N), 1261 (=C−O), 1226 (C−N). ^1^H NMR (CDCl_3_, δ ppm, *J*/Hz): 0.71 (s, 3H, 10-CH_3_); 0.85 (s, 3H, 8-CH_3_); 0.92 (s, 3H, 9-CH_3_); 1.27−1.46 (m, 2H, 5-CH_2_, 6-CH_2_); 1.52−1.61 (m, 2H, 3-CH_2_, 6-CH_2_); 1.67−1.83 (m, 2H, 5-CH_2_, 4-CH); 2.01−2.11 (m, 1H, 3-CH_2_); 2.37 (s, 3H, N-COCH_3_); 2.94 (s, 6H, C(23)-N(CH_3_)_2_); 3.28−3.34 (m, 2H, 18-CH_2_, 2-CH); 3.77−3.83 (m, 1H, 18-CH_2_); 4.59 (d, *J* = 4.8 Hz, 2H, 1′-CH_2_); 5.36 (d, *J* = 10.8 Hz, 1H, 3′-CH_2_(H*cis*)); 5.47−5.56 (m, 2H, 3′-CH_2_(H*trans*), 19-CH); 6.06−6.13 (m, 1H, 2′-CH); 6.55 (s, 1H, 13-CH); 6.69−6.73 (m, 2H, 21-CH, 25-CH); 7.14−7.19 (m, 3H, 22-CH, 24-CH, 16-CH); 10.39 (s, 1H, C(14)-OH). ^13^C NMR (CDCl_3_, δ ppm): 12.3 (10-CH_3_); 20.1 (8-CH_3_); 21.5 (9-CH_3_); 22.1 (N-COCH_3_); 27.4 (5-CH_2_); 34.1 (3-CH_2_); 39.6 (6-CH_2_); 40.5 (C(23)-N(CH_3_)_2_); 42.8 (18-CH_2_); 44.2 (2-CH); 44.3 (4-CH); 49.1 (7-C); 51.1 (1-C); 57.9 (19-CH-N); 68.8 (1′-CH_2_); 99.8 (13-CH); 107.3 (11-C); 112.7 (22-CH, 24-CH); 117.5 (3-CH_2′_); 124.1 (15-C); 126.7 (21-CH, 25-CH); 127.6 (16-CH); 129.1 (20-C); 132.8 (2′-CH); 150.13 (23-C); 156.8 (17-C=N); 160.7 and 167.3 (12-C) and (14-C); 199.8 (C=O). ESI-MS *m*/*z*: found 516.81 [M + H]^+^, calcd. for C_32_H_42_N_3_O_3_ 516.69.

*1-(5-(2-Methoxyphenyl)-3-(4′-allyloxy-2′-hydroxy-5′-isobornylphenyl)-4,5-dihydro-(1H)-pyrazole-1-yl)etanone* (**7e**). Gray-yellow powder; 96% yield; m.p. 70–71 °C. IR (KBr), ν/cm^−1^ 3427 (OH), 1668 (C=O), 1625 (C=N), 1259 (=C−O), 1239 (C−N), 1188 (O−CH_3_). ^1^H NMR (CDCl_3_, δ ppm, *J*/Hz): 0.71 (s, 3H, 10-CH_3_); 0.86 (s, 3H, 8-CH_3_); 0.93 (s, 3H, 9-CH_3_); 1.21−1.29 (m, 2H, 5-CH_2_, 6-CH_2_); 1.56−1.65 (m, 2H, 3-CH_2_, 6-CH_2_); 1.72−1.85 (m, 2H, 5-CH_2_, 4-CH); 2.05−2.15 (m, 1H, 3-CH_2_); 2.43 (s, 3H, N-COCH_3_); 3.05−3.18 (m, 1H, 18-CH_2_); 3.27 (t, *J* = 8.9 Hz, 1H, 2-CH); 3.72−3.98 (br.s, 4H, 18-CH_2_, C(21)-OCH_3_); 4.59 (d, *J* = 4.8 Hz, 2H, 1′-CH_2_); 5.35 (d, *J* = 11 Hz, 1H, 3′-CH_2_(H*cis*)); 5.52 (d, *J* = 16.8 Hz, 1H, 3′-CH_2_(H*trans*)); 5.76−5.81 (m, 1H, 19-CH); 6.07−6.12 (m, 1H, 2′-CH); 6.54 (s, 1H, 13-CH); 6.93 (d, *J* = 9 Hz, 2H, 22-CH, 25-CH); 7.06−7.09 (m, 2H, 24-CH, 16-CH); 7.27−7.29 (m, 1H, 23-CH); 10.44 (s, 1H, C(14)-OH). ^13^C NMR (CDCl_3_, δ ppm): 12.3 (10-CH_3_); 20.3 (8-CH_3_); 21.5 (9-CH_3_); 22.2 (N-COCH_3_); 27.4 (5-CH_2_); 34.2 (3-CH_2_); 39.3 (6-CH_2_); 41.7 (18-CH_2_); 44.3 (2-CH); 45.6 (4-CH); 48.1 (7-C); 49.5 (1-C); 54.1 (19-CH-N); 57.9 (C(21)-OCH_3_); 68.8 (1′-CH_2_); 99.8 (13-CH); 107.3 (11-C); 111.1 (22-CH, 25-CH); 117.5 (3′-CH_2_); 120.8 (24-CH); 122.2 (15-C); 124.2 (16-CH); 127.5 (20-C); 127.8 (23-CH); 132.8 (2′-CH); 156.1 (21-C); 156.3 (17-C=N); 157.5 and 160.6 (12-C) and (14-C); 199.8 (C=O). ESI-MS *m*/*z*: found 503.71, [M + H]^+^, calcd. for C_31_H_39_N_2_O_4_ 503.64.

*1-(5-(3-Methoxyphenyl)-3-(4′-allyloxy-2′-hydroxy-5′-isobornylphenyl)-4,5-dihydro-(1H)-pyrazole-1-yl)etanone* (**7f**). Gray-yellow powder; 91% yield; m.p. 64–65 °C. IR (KBr), ν/cm^−1^ 3429 (OH), 1668 (C=O), 1625 (C=N), 1259 (=C−O), 1247 (C−N), 1189 (O−CH_3_). ^1^H NMR (CDCl_3_, δ ppm, *J*/Hz): 0.71 (s, 3H, 10-CH_3_); 0.85 (s, 3H, 8-CH_3_); 0.92 (s, 3H, 9-CH_3_); 1.21−1.38 (m, 2H, 5-CH_2_, 6-CH_2_); 1.52−1.64 (m, 2H, 3-CH_2_, 6-CH_2_); 1.73−1.86 (m, 2H, 5-CH_2_, 4-CH); 2.03−2.12 (m, 1H, 3-CH_2_); 2.40 (s, 3H, N-COCH_3_); 3.25−3.31 (m, 2H, 18-CH_2_, 2-CH); 3.72−3.96 (br.s, 4H, 18-CH_2_, C(22)-OCH_3_); 4.59 (d, *J* = 4.7 Hz, 2H, 1′-CH_2_); 5.36 (d, *J* = 10.9 Hz, 1H, 3′-CH_2_(H*cis*)); 5.50−5.55 (m, 2H, 3′-CH_2_(H*trans*), 19-CH); 6.06−6.11 (m, 1H, 2′-CH); 6.55 (s, 1H, 13-CH); 6.82−6.86 (m, 3H, 23-CH, 24-CH, 25-CH); 7.10 (s, 1H, 16-CH); 7.29 (s, 1H, 21-CH); 10.31 (s, 1H,C(14)-OH). ^13^C NMR (CDCl_3_, δ ppm): 12.3 (10-CH_3_); 20.1 (8-CH_3_); 21.4 (9-CH_3_); 22.1 (N-COCH_3_); 27.4 (5-CH_2_); 33.9 (3-CH_2_); 39.6 (6-CH_2_); 42.6 (18-CH_2_); 44.2 (2-CH); 45.6 (4-CH); 48.1 (7-C); 49.7 (1-C); 54.2 (C(22)-OCH_3_); 58.1 (19-CH-N); 68.8 (1′-CH_2_); 99.9 (13-CH); 106.8 (11-C); 111.8 (24-CH); 112.9 (25-CH); 117.7 (3′-CH_2_); 117.9 (23-CH); 119.8 (15-C); 127.6 (16-CH); 130.13 (21-CH); 132.7 (2′-CH); 133.1 (20-C); 143.1 (22-C); 154.2 (17-C=N); 157.4 and 160.1 (12-C) и (14-C); 199.8 (C=O). ESI-MS *m*/*z*: found 503.66, [M + H]^+^, calcd. for C_31_H_39_N_2_O_4_ 503.64.

*1-(5-(4-Methoxyphenyl)-3-(4′-allyloxy-2′-hydroxy-5′-isobornylphenyl)-4,5-dihydro-(1H)-pyrazole-1-yl)etanone* (**7g**). Gray-yellow powder; 90% yield; m.p. 61–62 °C. IR (KBr), ν/cm^−1^ 3415 (OH), 1667 (C=O), 1626 (C=N), 1255 (=C−O), 1240 (C−N), 1184 (O−CH_3_). ^1^H NMR (CDCl_3_, δ ppm, *J*/Hz): 0.72 (s, 3H, 10-CH_3_); 0.85 (s, 3H, 8-CH_3_); 0.92 (s, 3H, 9-CH_3_); 1.29−1.48 (m, 2H, 5-CH_2_, 6-CH_2_); 1.51−1.61 (m, 2H, 3-CH_2_, 6-CH_2_); 1.72−1.83 (m, 2H, 5-CH_2_, 4-CH); 2.04−2.11 (m, 1H, 3-CH_2_); 2.38 (s, 3H, N-COCH_3_); 3.19−3.39 (m, 2H, 18-CH_2_, 2-CH); 3.72−3.91 (br.s, 4H, 18-CH_2_, C(23)-OCH_3_); 4.60 (d, *J* = 4.8 Hz, 2H, 1′-CH_2_); 5.35 (d, *J* = 10.8 Hz, 1H, 3′-CH_2_(H*cis*)); 5.50−5.55 (m, 2H, 3′-CH_2_(H*trans*), 19-CH); 6.07−6.13 (m, 1H, 2′-CH); 6.56 (s, 1H, 13-CH); 6.69−6.71 (m, 2H, 22-CH, 24-CH); 7.13 (s, 1H, 16-CH); 7.19−7.21 (m, 2H, 25-CH, 21-CH,); 10.33 (s, 1H, C(14)-OH). ^13^C NMR (CDCl_3_, δ ppm): 12.3 (10-CH_3_); 20.1 (8-CH_3_); 21.5 (9-CH_3_); 22.1 (N-COCH_3_); 27.4 (5-CH_2_); 34.1 (3-CH_2_); 39.6 (6-CH_2_); 42.7 (18-CH_2_); 44.3 (2-CH); 45.6 (4-CH); 48.1 (7-C); 49.5 (1-C); 54.3 (C(23)-OCH_3_); 57.8 (19-CH-N); 68.8 (1′-CH_2_); 99.9 (13-CH); 107.1 (11-C); 114.4 (22-CH, 24-CH); 117.5 (3′-CH_2_); 120.5 (15-C); 127 (21-CH, 25-CH); 127.5 (16-CH); 132.7 (2′-CH); 133.7 (20-C); 139.4 (23-C); 157.4 (17-C=N); 163.2 and 167.5 (12-C) and (14-C); 200.1 (C=O). ESI-MS *m*/*z*: found 503.62, [M + H]^+^, calcd. for C_31_H_39_N_2_O_4_ 503.64.

*1-(5-(2,3-Dimethoxyphenyl)-3-(4′-allyloxy-2′-hydroxy-5′-isobornylphenyl)-4,5-dihydro-(1H)-pyrazole-1-yl)etanone* (**7h**). Gray-yellow powder; 90% yield; m.p. 63–64 °C. IR (KBr), ν/cm^−1^ 3433 (OH), 1668 (C=O), 1627 (C=N), 1265 (=C−O), 1226 (C−N), 1188 (O−CH_3_). ^1^H NMR (CDCl_3_, δ ppm, *J*/Hz): 0.67 (s, 3H, 10-CH_3_); 0.80 (s, 3H, 8-CH_3_); 0.88 (s, 3H, 9-CH_3_); 1.16−1.25 (m, 2H, 5-CH_2_, 6-CH_2_); 1.31−1.34 (m, 2H, 3-CH_2_, 6-CH_2_); 1.43−1.71 (m, 2H, 5-CH_2_, 4-CH); 1.98−2.10 (m, 1H, 3-CH_2_); 2.37 (s, 3H, N-COCH_3_); 3.02−3.33 (m, 2H, 18-CH_2_, 2-CH); 3.72−3.98 (m, 7H, 18-CH_2_, C(21)-OCH_3_, C(22)-OCH_3_); 4.55 (d, *J* = 4.9 Hz, 2H, 1′-CH_2_); 5.31 (d, *J* = 11.2 Hz, 1H, 3′-CH_2_(H*cis*)); 5.49 (d, *J* = 16.4 Hz, 1H, 3′-CH_2_(H*trans*)); 5.68−5.72 (m, 1H, 19-CH); 6.05−6.09 (m, 1H, 2′-CH); 6.50 (s, 1H, 13-CH); 6.68−6.77 (m, 1H, 23-CH); 6.81−6.92 (m, 1H, 24-CH); 6.93−7.04 (m, 1H, 25-CH); 7.06 (s, 1H, 16-CH); 10.36 (s, 1H, C(14)-OH). ^13^C NMR (CDCl_3_, δ ppm): 12.3 (10-CH_3_); 20.5 (8-CH_3_); 21.4 (9-CH_3_); 22.1 (N-COCH_3_); 27.4 (5-CH_2_); 34.3 (3-CH_2_); 39.5 (6-CH_2_); 42.3 (18-CH_2_); 44.3 (2-CH); 45.5 (4-CH); 48.1 (7-C); 49.5 (1-C); 54.3 (19-CH-N); 55.8 (C(22)-OCH_3_); 60.4 (C(21)-OCH_3_); 68.8 (1′-CH_2_); 99.8 (13-CH); 107.2 (11-C); 111.9 (24-CH); 117.5 (3′-CH_2_); 118.3 (23-CH); 124.4 (15-C); 124.9 (25-CH); 127.7 (16-CH); 132.8 (2′-CH); 135.2 (20-C); 152.9 (22-C); 157.3 (21-C); 157.4 (17-C=N); 160.7 and 167.5 (12-C) and (14-C); 198.6 (C=O). ESI-MS *m*/*z*: found 533.60, [M + H]^+^, calcd. for C_32_H_41_N_2_O_5_ 533.67.

*1-(5-(3,4-Dimethoxyphenyl)-3-(4′-allyloxy-2′-hydroxy-5′-isobornylphenyl)-4,5-dihydro-(1H)-pyrazole-1-yl)etanone* (**7i**). Gray-yellow powder; 99% yield; m.p. 71–72 °C. IR (KBr), ν/cm^−1^ 3431 (OH), 1669 (C=O), 1626 (C=N), 1265 (=C−O), 1221 (C−N), 1186 (O−CH_3_). ^1^H NMR (CDCl_3_, δ ppm, *J*/Hz): 0.72 (s, 3H, 10-CH_3_); 0.83 (s, 3H, 8-CH_3_); 0.92 (s, 3H, 9-CH_3_); 1.19−1.57 (m, 2H, 5-CH_2_, 6-CH_2_); 1.61−1.72 (m, 2H, 3-CH_2_, 6-CH_2_); 1.79−1.92 (m, 2H, 5-CH_2_, 4-CH); 2.01−2.16 (m, 1H, 3-CH_2_); 2.40 (s, 3H, N-COCH_3_); 3.18−3.37 (m, 2H, 18-CH_2_, 2-CH); 3.79−4.02 (m, 7H, 18-CH_2_, C(22)-OCH_3_, C(23)-OCH_3_); 4.59 (d, *J* = 4.7 Hz, 2H, 1′-CH_2_); 5.31 (d, *J* = 11 Hz, 1H, 3′-CH_2_(H*cis*)); 5.48−5.55 (m, 2H, 3′-CH_2_(H*trans*), 19-CH); 6.05−6.16 (m, 1H, 2′-CH); 6.55 (s, 1H, 13-CH); 6.61−6.85 (m, 3H, 21-CH, 24-CH, 25-CH); 7.13 (s, 1H, 16-CH); 10.34 (s, 1H, C(14)-OH). ^13^C NMR (CDCl_3_, δ ppm): 12.3 (10-CH_3_); 20.1 (8-CH_3_); 21.5 (9-CH_3_); 22.1 (N-COCH_3_); 27.4 (5-CH_2_); 34.2 (3-CH_2_); 39.5 (6-CH_2_); 42.8 (18-CH_2_); 44.2 (2-CH); 45.6 (4-CH); 48.1 (7-C); 49.5 (1-C); 55.9 (C(22)-OCH_3_, C(23)-OCH_3_); 58.1 (19-CH-N); 68.8 (1′-CH_2_); 99.9 (13-CH); 107.1 (11-C); 109.3 (24-CH); 111. (25-CH); 117.5 (3′-CH_2_); 117.9 (21-CH); 125.3 (15-C); 127.6 (16-CH); 132.7 (2′-CH); 134.1 (20-C); 148.7 (23-C); 149.1 (22-C); 156.8 (17-C=N); 160.8 and 167.6 (12-C) and (14-C); 199.7 (C=O). ESI-MS *m*/*z*: found 533.73, [M + H]^+^, calcd. for C_32_H_41_N_2_O_5_ 533.67.

*1-(5-(2,4,6-Trimethoxyphenyl)-3-(4′-allyloxy-2′-hydroxy-5′-isobornylphenyl)-4,5-dihydro-(1H)-pyrazole-1-yl)etanone* (**7j**). Yellow oil; 75% yield. IR (KBr), ν/cm^−1^ 3427 (OH), 1658 (C=O), 1600 (C=N), 1261 (=C−O), 1232 (C−N), 1199 (O−CH_3_). ^1^H NMR (CDCl_3_, δ ppm, *J*/Hz): 0.72 (s, 3H, 10-CH_3_); 0.83 (s, 3H, 8-CH_3_); 0.92 (s, 3H, 9-CH_3_); 1.29−1.58 (m, 2H, 5-CH_2_, 6-CH_2_); 1.62−1.74 (m, 2H, 3-CH_2_, 6-CH_2_); 1.79−1.86 (m, 2H, 5-CH_2_, 4-CH); 2.01−2.13 (m, 1H, 3-CH_2_); 2.28 (s, 3H, N-COCH_3_); 3.21−3.29 (m, 2H, 18-CH_2_, 2-CH); 3.57−4.01 (m, 10H,18-CH_2_, C(21)-OCH_3_, C(23)-OCH_3_, C(25)-OCH_3_); 4.59 (d, *J* = 4.8 Hz, 2H, 1′-CH_2_); 5.35 (d, *J* = 10.9 Hz, 1H, 3′-CH_2_(H*cis*)); 5.53 (d, *J* = 15.7 Hz, 1H, 3′-CH_2_(H*trans*)); 5.59−6.16 (m, 4H, 2′-CH, 19-CH, 24-CH, 22-CH); 6.56 (s, 1H, 13-CH); 7.15 (s, 1H, 16-CH); 10.58 (s, 1H, C(14)-OH). ^13^C NMR (CDCl_3_, δ ppm): 12.3 (10-CH_3_); 20.1 (8-CH_3_); 21.5 (9-CH_3_); 22.1 (N-COCH_3_); 27.4 (5-CH_2_); 34.2 (3-CH_2_); 39.5 (6-CH_2_); 40.3 (18-CH_2_); 44.2 (2-CH); 45.6 (4-CH); 47.9 (7-C); 49.3 (19-CH-N); 49.5 (1-C); 55.3 (C(21)-OCH_3_, C(23)-OCH_3_, C(25)-OCH_3_); 68.7 (1′-CH_2_); 91.3 (22-CH, 24-CH); 99.7 (13-CH); 107.6 (11-C); 117.3 (3′-CH_2_); 125.6 (15-C); 127.4 (16-CH); 132.9 (2′-CH); 140.1 (20-C); 149.2 (23-C); 156.9 (25-C); 157.1 (21-C); 159.1 (17-C=N); 160.9 and 167.1 (12-C) and (14-C); 200.1 (C=O). ESI-MS *m*/*z*: found 563.81, [M + H]^+^, calcd. for C_33_H_43_N_2_O_6_ 563.70.

*1-(5-(3,4,5-Trimethoxyphenyl)-3-(4′-allyloxy-2′-hydroxy-5′-isobornylphenyl)-4,5-dihydro-(1H)-pyrazole-1-yl)etanone* (**7k**). Gray-yellow powder; 98% yield; m.p. 73–74 °C. IR (KBr), ν/cm^−1^ 3431 (OH), 1670 (C=O), 1593 (C=N), 1261 (=C−O), 1238 (C−N), 1188 (O−CH_3_). ^1^H NMR (CDCl_3_, δ ppm, *J*/Hz): 0.71 (s, 3H, 10-CH_3_); 0.84 (s, 3H, 8-CH_3_); 0.92 (s, 3H, 9-CH_3_); 1.29−1.48 (m, 2H, 5-CH_2_, 6-CH_2_); 1.59−1.71 (m, 2H, 3-CH_2_, 6-CH_2_); 1.79−1.91 (m, 2H, 5-CH_2_, 4-CH); 2.01−2.17 (m, 1H, 3-CH_2_); 2.42 (s, 3H, N-COCH_3_); 3.25−3.31 (m, 2H, 18-CH_2_, 2-CH); 3.79−4.01 (m, 10H, 18-CH_2_, C(22)-OCH_3_, C(23)-OCH_3_, C(24)-OCH_3_); 4.59 (d, *J* = 4.7 Hz, 2H, 1′-CH_2_); 5.35 (d, *J* = 10.8 Hz, 1H, 3′-CH_2_(H*cis*)); 5.47−5.55 (m, 2H, 3′-CH_2_(H*trans*), 19-CH); 6.07−6.13 (m, 1H, 2′-CH); 6.46 (s, 1H, 25-CH); 6.49 (s, 1H, 21-CH); 6.56 (s, 1H, 13-CH); 7.12 (s, 1H, 16-CH); 10.31 (s, 1H, C(14)-OH). ^13^C NMR (CDCl_3_, δ ppm): 12.2 (10-CH_3_); 20.1 (8-CH_3_); 21.5 (9-CH_3_); 22.1 (N-COCH_3_); 27.4 (5-CH_2_); 34.2 (3-CH_2_); 39.5 (6-CH_2_); 42.8 (18-CH_2_); 44.2 (2-CH); 45.6 (4-CH); 48 (7-C); 49.5 (1-C); 56.22 (C(24)-OCH_3_, C(22)-OCH_3_); 58.2 (19-CH-N); 60.8 (C(23)-OCH_3_); 68.8 (1′-CH_2_); 99.9 (13-CH); 102.55 (25-CH); 102.8 (21-CH); 107.1 (11-C); 117.5 (3′-CH_2_); 123.6 (15-C); 127.5 (16-CH); 132.7 (2′-CH); 137.2 (20-C); 153.8 (22-C, 23-C, 24-C); 156.8 (17-C=N); 160.9 and 167.7 (12-C) and (14-C); 199.7 (C=O). ESI-MS *m*/*z*: found 563.68, [M + H]^+^, calcd. for C_33_H_43_N_2_O_6_ 563.70.

*1-(5-(3-Nitrophenyl)-3-(2′,4′-diallyloxy-5′-isobornylphenyl)-4,5-dihydro-(1H)-pyrazole-1-yl)etanone* (**9a**). Gray-yellow powder; 85% yield; m.p. 58–60 °C. IR (KBr), ν/cm^−1^ 1660 (C=O), 1610 (C=N), 1350 (N−O), 1259 (=C−O), 1220 (C−N). ^1^H NMR (CDCl_3_, δ ppm, *J*/Hz): 0.75 (s, 3H, 10-CH_3_); 0.89 (s, 3H, 8-CH_3_); 0.97 (s, 3H, 9-CH_3_) 1.29−1.56 (m, 2H, 5-CH_2_, 6-CH_2_); 1.61−1.72 (m, 2H, 3-CH_2_, 6-CH_2_); 1.79−1.95 (m, 2H, 5-CH_2_, 4-CH); 2.19−2.31 (m, 1H, 3-CH_2_); 2.44 (s, 3H, N-COCH_3_); 3.26−3.48 (m, 2H, 18-CH_2_ 2-CH); 3.89−3.98 (m, 1H, 18-CH_2_); 4.53−4.67 (m, 4H, 1′-CH_2_, 1′′-CH_2_); 5.22−5.43 (m, 3H, 3′-CH_2_(H*cis*), 3′′-CH_2_(H*cis*), 19-CH); 5.49−5.69 (m, 2H, 3′-CH_2_(H*trans*), 3′′-CH_2_(H*trans*)); 6.02−6.12 (m, 2H, 2′-CH, 2′′-CH); 6.44 (s, 1H, 13-CH); 7.49−7.56 (m, 1H, 24-CH); 7.61−7.65 (m, 1H, 25-CH); 7.94 (d, *J* = 16.5 Hz, 1H, 16-CH); 8.12−8.20 (m, 2H, 21-CH, 23-CH). ^13^C NMR (CDCl_3_, δ ppm): 12.4 (10-CH_3_); 20.2 (8-CH_3_); 21.6 (9-CH_3_); 21.9 (N-COCH3); 27.5 (5-CH_2_); 34.3 (3-CH_2_); 40.3 (6-CH_2_); 44.4 (2-CH); 45.4 (4-CH); 45.7 (18-CH_2_); 48.1 (7-C); 49.6 (1-C); 59.2 (19-CH-N); 68.8 (1′-CH_2_); 69.8 (1′′-CH_2_); 97.1 (13-CH); 111.9 (11-C); 117.4 (3′-CH_2_); 118.3 (3′′-CH_2_); 121 (23-CH); 122.5 (21-CH); 125.7 (15-C); 128.7 (16-CH); 129.7 (24-CH, 25-CH); 132.8 (2′-CH, 2′′-CH); 144.4 (20-C); 148.6 (22-C); 154.1 (17-C=N); 160.5 and 168.9 (12-C) and (14-C); 210.1 (C=O). ESI-MS *m*/*z*: found 558.67 [M + H]^+^, calcd. for C_33_H_40_N_3_O_5_ 558.68.

*1-(5-(4-Clorophenyl)-3-(2′,4′-diallyloxy-5′-isobornylphenyl)-4,5-dihydro-(1H)-pyrazole-1-yl)etanone* (**9b**). Gray-yellow powder; 76% yield; m.p. 64–65 °C. IR (KBr), ν/cm^−1^ 1662 (C=O), 1610 (C=N), 1259 (=C−O), 1219 (C−N), 1016 (Ar−Cl). ^1^H NMR (CDCl_3_, δ ppm, *J*/Hz): 0.75 (s, 3H, 10-CH_3_); 0.88 (s, 3H, 8-CH_3_); 0.96 (s, 3H, 9-CH_3_); 1.29−1.58 (m, 2H, 5-CH_2_, 6-CH_2_); 1.49−1.64 (m, 2H, 3-CH_2_, 6-CH_2_); 1.82−1.96 (m, 2H, 5-CH_2_, 4-CH_2_); 2.19−2.29 (m, 1H, 3-CH_2_); 2.4 (s, 3H, N-COCH_3_); 3.25−3.41 (m, 2H, 18-CH_2_, 2-CH); 3.81−3.90 (m, 1H, 18-CH_2_); 4.51−4.68 (m, 4H, 1′-CH_2_, 1′′-CH_2_); 5.32−5.38 (m, 3H, 3′-CH_2_(H*cis*), 3′′-CH_2_(H*cis*), 19-CH); 5.52 (d, *J* = 17 Hz, 2H, 3′-CH_2_(H*trans*), 3′′-CH_2_(H*trans*)); 6.03−6.10 (m, 2H, 2′-CH, 2′′-CH); 6.43 (s, 1H, 13-CH); 7.21 (d, *J* = 8.1 Hz, 2H, 21-CH, 25-CH); 7.28 (d, *J* = 8 Hz, 2H, 22-CH, 24-CH); 7.98 (d, *J* = 16.5 Hz, 1H, 16-CH). ^13^C NMR (CDCl_3_, δ ppm): 12.4 (10-CH_3_); 20.1 (8-CH_3_); 21.5 (9-CH_3_); 21.9 (N-COCH3); 27.5 (5-CH_2_); 34.2 (3-CH_2_); 39.6 (6-CH_2_); 44.4 (2-CH); 45.5 (18-CH_2_); 45.6 (4-CH); 48.1 (7-C); 49.6 (1-C); 59.2 (19-CH-N); 68.8 (1′-CH_2_); 69.9 (1′′-CH_2_); 97.1 (13-CH); 111.3 (11-C); 117.4 (3′-CH_2_); 118.3 (3′′-CH_2_); 125.5 (15-C); 127.2 (21-CH, 25-CH) 128.5 (16-CH, 22-CH, 24-CH); 132.9 (2′-CH, 2′′-CH); 133.1 (20-C); 140.9 (23-C); 154.2 (17-C=N); 160.4 and 168.7 (12-C) and (14-C); 209.6 (C=O). ESI-MS *m*/*z*: found 548.18 [M + H]^+^, calcd. for C_33_H_40_ClN_2_O_3_ 548.14.

*1-(5-(4-Bromophenyl)-3-(2′,4′-diallyloxy-5′-isobornylphenyl)-4,5-dihydro-(1H)-pyrazole-1-yl)etanone* (**9c**). Yellow powder; 91% yield; m.p. 60–61 °C. IR (KBr), ν/cm^−1^ 1662 (C=O), 1608 (C=N), 1259 (=C−O), 1220 (C−N). ^1^H NMR (CDCl_3_, δ ppm, *J*/Hz): 0.75 (s, 3H, 10-CH_3_); 0.88 (s, 3H, 8-CH_3_); 0.96 (s, 3H, 9-CH_3_); 1.29−1.49 (m, 2H, 5-CH_2_, 6-CH_2_); 1.60−1.64 (m, 2H, 3-CH_2_, 6-CH_2_); 1.72−1.96 (m, 2H, 5-CH_2_, 4-CH); 2.12−2.33 (m, 1H, 3-CH_2_); 2.42 (s, 3H, N-COCH_3_); 3.19−3.42 (m, 2H, 18-CH_2_, 2-CH); 3.76−4.01 (m, 1H, 18-CH_2_); 4.50−4.65 (m, 4H, 1′-CH_2_, 1′′-CH_2_); 5.28−5.40 (m, 3H, 3′-CH_2_(H*cis*), 3′′-CH_2_(H*cis*), 19-CH); 5.52 (d, *J* = 16.8 Hz, 2H, 3′-CH_2_(H*trans*), 3′′-CH_2_(H*trans*)); 6.06−6.10 (m, 2H, 2′-CH, 2′′-CH); 6.43 (s, 1H, 13-CH); 7.15 (d, *J* = 8.3 Hz, 2H, 21-CH, 25-CH); 7.45 (d, *J* = 8.3 Hz, 2H, 22-CH, 24-CH); 7.11 (d, *J* = 16.5 Hz, 1H, 16-CH). ^13^C NMR (CDCl_3_, δ ppm): 12.5 (10-CH_3_); 20.1 (8-CH_3_); 21.6 (9-CH_3_); 22.5 (N-COCH_3_); 27.5 (5-CH_2_); 34.2 (3-CH_2_); 39.6 (6-CH_2_); 44.3 (2-CH); 44.4 (4-CH); 45.5 (18-CH_2_); 48.1 (7-C); 49.6 (1-C); 59.3 (19-CH-N); 68.8 (1′-CH_2_); 69.8 (1′′-CH_2_); 97.1 (13-CH); 111.2 (11-C); 117.4 (3′-CH_2_); 118.3 (3′′-CH_2_); 127.4 (15-C); 127.7 (21-CH, 25-CH); 131.8 (16-CH, 22-CH, 24-CH); 132.9 (2′-CH,2′′-CH); 136.5 (20-C); 141.2 (23-C); 157.1 (17-C=N); 164.1 and 168.5 (12-C) and (14-C); 203.2 (C=O). ESI-MS *m*/*z*: found 592.28 [M + H]^+^, calcd. for C_33_H_40_BrN_2_O_3_ 592.58.

*1-(5-(4-Dimethylaminophenyl)-3-(2′,4′-diallyloxy-5′-isobornylphenyl)-4,5-dihydro-(1H)-pyrazole-1-yl)etanone* (**9d**). Yellow oil; 68% yield. IR (KBr), ν/cm^−1^ 1658 (C=O), 1612 (C=N), 1259 (=C−O), 1219 (C−N). ^1^H NMR (CDCl_3_, δ ppm, *J*/Hz): 0.75 (s, 3H, 10-CH_3_); 0.87(s, 3H, 8-CH_3_); 0.97 (s, 3H, 9-CH_3_); 1.29−1.55 (m, 2H, 5-CH_2_, 6-CH_2_); 1.60−1.69 (m, 2H, 3-CH_2_, 6-CH_2_); 1.72−1.95 (m, 2H, 5-CH_2_, 4-CH); 2.15−2.34 (m, 1H, 3-CH_2_); 2.39 (s, 3H, N-COCH_3_); 2.93 (s, 6H, C(23)-N(CH_3_)_2_); 3.28−3.44 (m, 2H, 18-CH_2_, 2-CH); 3.73−3.91 (m, 1H, 18-CH_2_); 4.57 (d, *J* = 4.9 Hz, 4H, 1′-CH_2_, 1′′-CH_2_); 5.28−5.55 (m, 5H, 3′-CH_2_(H*cis*), 3′′-CH_2_(H*cis*), 19-CH, 3′-CH_2_(H*trans*), 3′′-CH_2_(H*trans*)); 6.07−6.10 (m, 2H, 2′-CH, 2′′-CH); 6.44 (s, 1H, 13-CH); 6.71 (d, *J* = 8.1 Hz, 2H, 21-CH, 25-CH); 7.17−7.20 (m, 2H, 22-CH, 24-CH); 7.95 (d, *J* = 16.5 Hz, 1H, 16-CH). ^13^C NMR (CDCl_3_, δ ppm): 12.4 (10-CH_3_); 20.2 (8-CH_3_); 21.6 (9-CH_3_); 22.1 (N-COCH3); 27.5 (5-CH_2_); 34.2 (3-CH_2_); 39.6 (6-CH_2_); 40.7 (C(23)-N(CH_3_)_2_); 44.4 (2-CH); 44.6 (4-CH); 45.4 (18-CH_2_); 48.1 (7-C); 49.6 (1-C); 59.3 (19-CH-N); 68.8 (1′-CH_2_); 69.8 (1′′-CH_2_); 97.2 (13-CH); 111.1 (11-C); 112.8 (22-CH, 24-CH); 117.3 (3′-CH_2_); 118.1 (3′′-CH_2_); 125.4 (15-C); 126.9 (21-CH, 25-CH); 128.5 (16-CH); 130.4 (20-C); 133 (2′-CH, 2′′-CH); 137.5 (23-C); 150 (17-C=N); 154.3 and 160.1 (12-C) and (14-C); 197.5 (C=O). ESI-MS *m*/*z*: found 556.77 [M + H]^+^, calcd. for C_35_H_46_N_3_O_3_ 556.75.

*1-(5-(2-Methoxyhenyl)-3-(2′,4′-diallyloxy-5′-isobornylphenyl)-4,5-dihydro-(1H)-pyrazole-1-yl)etanone* (**9e**). Yellow powder; 93% yield; m.p. 55–56 °C. IR (KBr), ν/cm^−1^ 1658 (C=O), 1608 (C=N), 1247 (=C−O), 1219 (C−N), 1192 (O−CH_3_). ^1^H NMR (CDCl_3_, δ ppm, *J*/Hz): 0.76 (s, 3H, 10-CH_3_); 0.89 (s, 3H, 8-CH_3_); 0.96 (s, 3H, 9-CH_3_); 1.30−1.49 (m, 2H, 5-CH_2_, 6-CH_2_); 1.64−1.68 (m, 2H, 3-CH_2_, 6-CH_2_); 1.69−1.89 (m, 2H, 5-CH_2_, 4-CH); 2.20−2.39 (m, 1H, 3-CH_2_); 2.46 (s, 3H, N-COCH_3_); 3.13−3.32 (m, 2H, 18-CH_2_, 2-CH); 3.71−4.09 (br.s, 4H, 18-CH_2_, C(21)-OCH_3_); 4.55 (d, *J* = 4.8 Hz, 4H, 1′-CH_2_, 1′′-CH_2_); 5.30−5.41 (m, 3H, 3′-CH_2_(H*cis*), 3′′-CH_2_(Hcis), 3′-CH_2_(H*trans*)); 5.52 (d, *J* = 16.7 Hz, 1H, 3′′-CH_2_(H*trans*)); 5.80 (d, *J* = 11 Hz, 1H, 19-CH); 6.04−6.10 (m, 2H, 2′-CH, 2′′-CH); 6.42 (s, 1H, 13-CH); 6.85−7.01 (m, 2H, 22-CH, 24-CH); 7.05−7.11 (m, 1H, 23-CH); 7.21−7.29 (m, 1H, 25-CH); 7.95 (d, *J* = 16.5 Hz, 1H, 16-CH). ^13^C NMR (CDCl_3_, δ ppm): 12.4 (10-CH_3_); 20.2 (8-CH_3_); 21.6 (9-CH_3_); 22 (N-COCH_3_); 27.5 (5-CH_2_); 34.2 (3-CH_2_); 39.6 (6-CH_2_); 44.3 (2-CH); 44.7 (18-CH_2_); 45.7 (4-CH); 48.1 (7-C); 49.6 (1-C); 55.4 (19-CH-N, C(22)-OCH_3_); 68.7 (1′-CH_2_); 69.9 (1′′-CH_2_); 97.2 (13-CH); 110.8 (24-CH); 111.8 (11-C); 117.3 (3′-CH_2_); 117.8 (3′′-CH_2_); 120.6 (22-CH); 125.3 (15-C); 125.8 (23-CH); 128.3 (16-CH, 25-CH); 129.8 (20-C); 133 (2′-CH, 2′′-CH); 154.9 (21-C); 156.2 (17-C=N); 160.1 and 168.6 (12-C) and (14-C); 200.3 (C=O). ESI-MS *m*/*z*: found 543.68, [M + H]^+^, calcd. for C_34_H_43_N_2_O_4_ 543.71.

*1-(5-(3-Methoxyhenyl)-3-(2′,4′-diallyloxy-5′-isobornylphenyl)-4,5-dihydro-(1H)-pyrazole-1-yl)etanone* (**9f**). Yellow-brown powder; 77% yield; m.p. 50–51 °C. IR (KBr), ν/cm^−1^ 1662 (C=O), 1609 (C=N), 1261 (=C−O), 1217 (C−N), 1192 (O−CH_3_). ^1^H NMR (CDCl_3_, δ ppm, *J*/Hz): 0.75 (s, 3H, 10-CH_3_); 0.88 (s, 3H, 8-CH_3_); 0.96 (s, 3H, 9-CH_3_); 1.29−1.57 (m, 2H, 5-CH_2_, 6-CH_2_); 1.60−1.68 (m, 2H, 3-CH_2_, 6-CH_2_); 1.81−1.89 (m, 2H, 5-CH_2_, 4-CH); 2.20−2.34 (m, 1H, 3-CH_2_); 2.43 (s, 3H, N-COCH_3_); 3.26−3.40 (m, 2H, 18-CH_2_, 2-CH); 3.75−4.01 (br.s, 4H, 18-CH_2_, C(22)-OCH_3_); 4.56 (d, *J* = 4.9 Hz, 4H, 1′-CH_2_, 1′′-CH_2_,); 5.33−5.55 (m, 5H, 3′-CH_2_(H*cis*), 3′′-CH_2_(H*cis*), 19-CH, 3′-CH_2_(H*trans*), 3′′-CH_2_(H*trans*)); 6.06−6.12 (m, 2H, 2′-CH, 2′′-CH); 6.43 (s, 1H, 13-CH); 6.78−6.89 (m, 3H, 21-CH, 23-CH, 25-CH); 7.24 (t, *J* = 8 Hz, 1H, 24-CH); 7.95 (d, *J* = 16.4 Hz, 1H, 16-CH). ^13^C NMR (CDCl_3_, δ ppm): 12.4 (10-CH_3_); 20.2 (8-CH_3_); 21.6 (9-CH_3_); 21.9 (N-COCH_3_); 27.5 (5-CH_2_); 34.2 (3-CH_2_); 39.6 (6-CH_2_); 44.4 (2-CH); 45.6 (4-CH); 45.7 (18-CH_2_); 48.1 (7-C); 49.6 (1-C); 55.2 (C(22)-OCH_3_); 59.7 (19-CH-N); 68.8 (1′-CH_2_); 69.8 (1′′-CH_2_); 97.2 (13-CH); 111.4 (25-CH); 111.6 (11-C); 112.8 (23-CH); 117.4 (3′-CH_2_); 117.9 (3′′-CH_2_); 118.1 (21-CH); 125.5 (15-C); 128.5 (16-CH); 129.8 (24-CH); 132.9 (2′-CH, 2′′-CH); 141.5 (20-C); 143.9 (22-C); 154.3 (17-C=N); 160.2 and 168.7 (12-C) and (14-C); 200.5 (C=O). ESI-MS *m*/*z*: found 543.79, [M + H]^+^, calcd. for C_34_H_43_N_2_O_4_ 543.71.

*1-(5-(4-Methoxyhenyl)-3-(2′,4′-diallyloxy-5′-isobornylphenyl)-4,5-dihydro-(1H)-pyrazole-1-yl)etanone* (**9g**). Yellow oil; 82% yield. IR (KBr), ν/cm^−1^ 1659 (C=O), 1610 (C=N), 1249 (=C−O), 1220 (C−N), 1184 (O−CH_3_). ^1^H NMR (CDCl_3_, δ ppm, *J*/Hz): 0.75 (s, 3H, 10-CH_3_); 0.88 (s, 3H, 8-CH_3_); 0.97 (s, 3H, 9-CH_3_); 1.29−1.46 (m, 2H, 5-CH_2_, 6-CH_2_); 1.60−1.67 (m, 2H, 3-CH_2_, 6-CH_2_); 1.89−1.95 (m, 2H, 5-CH_2_, 4-CH); 2.15−2.32 (m, 1H, 3-CH_2_); 2.42 (s, 3H, N-COCH_3_); 3.29−3.44 (m, 2H, 18-CH_2_, 2-CH); 3.75−4.01 (br.s, 4H, 18-CH_2_, C(23)-OCH_3_); 4.56 (d, *J* = 4.8 Hz, 4H, 1′-CH_2_, 1′′-CH_2_); 5.31−5.55 (m, 5H, 3′-CH_2_(H*cis*), 3′′-CH_2_(H*cis*), 19-CH, 3′-CH_2_(H*trans*), 3′′-CH_2_(H*trans*); 6.07−6.11 (m, 2H, 2′-CH, 2′′-CH); 6.44 (s, 1H, 13-CH); 6.87 (d, *J* = 8 Hz, 2H, 22-CH, 24-CH); 7.24 (d, *J* = 8.1 Hz, 2H, 21-CH, 25-CH); 7.95 (d, *J* = 16.5 Hz, 1H, 16-CH). ^13^C NMR (CDCl_3_, δ ppm): 12.4 (10-CH_3_); 20.2 (8-CH_3_); 21.6 (9-CH_3_); 21.8 (N-COCH_3_); 27.5 (5-CH_2_); 34.2 (3-CH_2_); 39.6 (6-CH_2_); 44.3 (2-CH); 44.5 (4-CH); 45.5 (18-CH_2_); 48.1 (7-C); 49.6 (1-C); 55.27 (C(23)-OCH_3_); 59.3 (19-CH-N); 68.8 (1′-CH_2_); 69.9 (1′′-CH_2_); 97.2 (13-CH); 110.7 (11-C); 114.1 (22-CH, 24-CH); 116.9 (3′-CH_2_); 118.2 (3′′-CH_2_); 125.5 (15-C); 127.2 (21-CH, 25-CH); 128.5 (16-CH); 132.9 (2′-CH, 2′′-CH); 134.5 (20-C); 154.6 (23-C); 156.4 (17-C=N); 165.1 and 168.8 (12-C) and (14-C); 201.3 (C=O). ESI-MS *m*/*z*: found 543.74, [M + H]^+^, calcd. for C_34_H_43_N_2_O_4_ 543.71.

*1-(5-(2,3-Dimethoxyhenyl)-3-(2′,4′-diallyloxy-5′-isobornylphenyl)-4,5-dihydro-(1H)-pyrazole-1-yl)etanone* (**9h**). White powder; 92% yield; m.p. 52–53 °C. IR (KBr), ν/cm^−1^ 1658 (C=O), 1608 (C=N), 1267 (=C−O), 1219 (C−N), 1190 (O−CH_3_). ^1^H NMR (CDCl_3_, δ ppm, *J*/Hz): 0.76 (s, 3H, 10-CH_3_); 0.89 (s, 3H, 8-CH_3_); 0.96 (s, 3H, 9-CH_3_); 1.29−1.41 (m, 2H, 5-CH_2_, 6-CH_2_); 1.60−1.67 (m, 2H, 3-CH_2_, 6-CH_2_); 1.79−1.96 (m, 2H, 5-CH_2_, 4-CH); 2.28−2.31 (m, 1H, 3-CH_2_); 2.42 (s, 3H, N-COCH_3_); 3.15−3.30 (m, 2H, 18-CH_2_, 2-CH); 3.81−4.05 (m, 7H, 18-CH_2_, C(21)-OCH_3_, C(22)-OCH_3_); 4.55 (d, *J* = 4.7 Hz, 4H, 1′-CH_2_, 1′′-CH_2_); 5.25−5.39 (m, 3H, 3′-CH_2_(H*cis*), 3′′-CH_2_(H*cis*), 3′-CH_2_(H*trans*)); 5.57 (d, *J* = 16.8 Hz, 1H, 3′′-CH_2_(H*trans*)); 5.71−5.80 (m, 1H, 19-CH); 5.96−6.13 (m, 2H, 2′-CH, 2′′-CH); 6.41 (s, 1H, 13-CH); 6.72−6.81 (m, 2H, 23-CH, 25-CH); 7.01 (t, *J* = 8.1 Hz, 1H, 24-CH); 7.97 (d, *J* = 16.6 Hz, 1H, 16-CH). ^13^C NMR (CDCl_3_, δ ppm): 12.4 (10-CH_3_); 20.2 (8-CH_3_); 21.6 (9-CH_3_); 21.9 (N-COCH_3_); 27.5 (5-CH_2_); 34.2 (3-CH_2_); 39.7 (6-CH_2_); 44.4 (2-CH); 45.3 (18-CH_2_); 45.7 (4-CH); 48.1 (7-C); 49.6 (1-C); 55.2 (C(22)-OCH_3_); 55.3 (19-CH-N); 60.3 (C(21)-OCH_3_); 68.8 (1′-CH_2_); 69.8 (1′′-CH_2_); 97.2 (13-CH); 111.5 (23-CH); 112.4 (11-C); 117.3 (3′-CH_2_); 118.2 (3′′-CH_2_); 118.7 (25-CH); 124.2 (24-CH); 125.3 (15-C); 128.5 (16-CH); 133 (2′-CH, 2′′-CH); 136.3 (20-C); 154.7 (22-C); 154.8 (21-C); 156.4 (17-C=N); 160.1 and 168.5 (12-C) and (14-C); 200.1 (C=O). ESI-MS *m*/*z*: found 573.74, [M + H]^+^, calcd. for C_35_H_45_N_2_O_5_ 573.73.

*1-(5-(3,4-Dimethoxyhenyl)-3-(2′,4′-diallyloxy-5′-isobornylphenyl)-4,5-dihydro-(1H)-pyrazole-1-yl)etanone* (**9i**). Yellow-brown powder; 99% yield; m.p. 50–51 °C. IR (KBr), ν/cm^−1^ 1658 (C=O), 1609 (C=N), 1259 (=C−O), 1235 (C−N), 1192 (O−CH_3_). ^1^H NMR (CDCl_3_, δ ppm, *J*/Hz): 0.76 (s, 3H, 10-CH_3_); 0.87 (s, 3H, 8-CH_3_); 0.95 (s, 3H, 9-CH_3_); 1.29−1.52 (m, 2H, 5-CH_2_, 6-CH_2_); 1.60−1.71 (m, 2H, 3-CH_2_, 6-CH_2_); 1.79−1.96 (m, 2H, 5-CH_2_, 4-CH); 2.19−2.31 (m, 1H, 3-CH_2_); 2.42 (s, 3H, N-COCH_3_); 3.25−3.43 (m, 2H, 18-CH_2_, 2-CH); 3.79−4.01 (m, 7H, 18-CH_2_, C(22)-OCH_3_, C(23)-OCH_3_); 4.56 (d, *J* = 4.9 Hz, 4H, 1′-CH_2_, 1′′-CH_2_); 5.27−5.54 (m, 5H, 3′-CH_2_(H*cis*), 3′′-CH_2_(H*cis*), 19-CH, 3′-CH_2_(H*trans*), 3′′-CH_2_(H*trans*)); 5.96−6.13 (m, 2H, 2′-CH, 2′′-CH); 6.44 (s, 1H, 13-CH); 6.82 (br.s, 3H, 21-CH, 24-CH, 25-CH); 7.95 (d, *J* = 16.7 Hz, 1H, 16-CH). ^13^C NMR (CDCl_3_, δ ppm): 12.4 (10-CH_3_); 20.1 (8-CH_3_); 21.5 (9-CH_3_); 21.9 (N-COCH_3_); 27.5 (5-CH_2_); 34.2 (3-CH_2_); 39.7 (6-CH_2_); 44.4 (2-CH); 45.6 (18-CH_2_); 45.7 (4-CH); 48.1 (7-C); 49.6 (1-C); 55.8 (C(22)-OCH_3_, C(23)-OCH_3_); 59.6 (19-CH-N); 68.8 (1′-CH_2_); 69.8 (1′′-CH_2_); 97.1 (13-CH); 109.2 (24-CH); 111.4 (25-CH); 112.2 (11-C); 117.3 (3′-CH_2_); 117.9 (21-CH); 118.2 (3′′-CH_2_); 125.4 (15-C); 128.5 (16-CH); 132.9 (2′-CH, 2′′-CH); 135.1 (20-C); 148.3 (23-C); 149.1 (22-C); 154.4 (17-C=N); 160.2 and 168.7 (12-C) and (14-C); 201.6 (C=O). ESI-MS *m*/*z*: found 573.59, [M + H]^+^, calcd. for C_35_H_45_N_2_O_5_ 573.73.

*1-(5-(2,4,6-Trimethoxyhenyl)-3-(2′,4′-diallyloxy-5′-isobornylphenyl)-4,5-dihydro-(1H)-pyrazole-1-yl)etanone* (**9j**). Yellow oil; 56% yield. IR (KBr), ν/cm^−1^ 1653 (C=O), 1610 (C=N), 1263 (=C−O), 1246 (C−N), 1199 (O−CH_3_). ^1^H NMR (CDCl_3_, δ ppm, *J*/Hz): 0.76 (s, 3H, 10-CH_3_); 0.87 (s, 3H, 8-CH_3_); 0.98 (s, 3H, 9-CH_3_); 1.29−1.63 (m, 2H, 5-CH_2_, 6-CH_2_); 1.61−1.72 (m, 2H, 3-CH_2_, 6-CH_2_); 1.81−1.96 (m, 2H, 5-CH_2_, 4-CH); 2.21−2.36 (m, 4H, 3-CH_2_, N-COCH_3_); 3.18−3.31 (m, 2H, 18-CH_2_, 2-CH); 3.58−4.05 (m, 10H, 18-CH_2_, C(21)-OCH_3_, C(23)-OCH_3_, C(25)-OCH_3_); 4.59 (d, *J* = 5 Hz, 4H, 1′-CH_2_, 1′′-CH_2_); 5.28 (d, *J* = 10 Hz, 1H, 3′-CH_2_(H*cis*)); 5.31 (d, *J* = 10.5 Hz, 1H, 3′′-CH_2_(H*cis*)); 5.41 (d, *J* = 16.3 Hz, 1H, 3′-CH_2_(H*trans*)); 5.52 (d, *J* = 16.7 Hz, 1H, 3′′-C(H*trans*)); 6.01−6.15 (m, 5H, 2′-CH, 2′′-CH, 19-CH, 24-CH, 22-CH); 6.45 (s, 1H, 13-CH); 7.96 (s, 1H, 16-CH). ^13^C NMR (CDCl_3_, δ ppm): 12.4 (10-CH_3_); 20.1 (8-CH_3_); 21.5 (9-CH_3_); 21.9 (N-COCH_3_); 27.7 (5-CH_2_); 34.2 (3-CH_2_); 39.7 (6-CH_2_); 42.9 (18-CH_2_); 44.4 (2-CH); 45.7 (4-CH); 49.7 (7-C); 49.9 (1-C); 50.6 (19-CH-N); 55.26 (C(21)-OCH_3_, C(23)-OCH_3_, C(25)-OCH_3_); 68.8 (1′-CH_2_); 69.9 (1′′-CH_2_); 91.4 (22-CH, 24-CH); 99.7 (13-CH); 111.3 (11-C); 117.2 (3′-CH_2_); 117.8 (3′′-CH_2_); 125.1 (15-C); 128.7 (16-CH); 133.2 (2′-CH, 2′′-CH); 141.2 (20-C); 154.3 (23-C); 156.2 (25-C, 21-C); 159.6 (17-C=N); 160.4 and 168.1 (12-C) and (14-C); 202.3 (C=O). ESI-MS *m*/*z*: found 603.52, [M + H]^+^, calcd. for C_36_H_47_N_2_O_6_ 603.76.

*1-(5-(3,4,5-Trimethoxyhenyl)-3-(2′,4′-diallyloxy-5′-isobornylphenyl)-4,5-dihydro-(1H)-pyrazole-1-yl)etanone* (**9k**). Yellow powder; 96% yield; m.p. 51–52 °C. IR (KBr), ν/cm^−1^ 1660 (C=O), 1608 (C=N), 1259 (=C−O), 1243 (C−N), 1190 (O−CH_3_). ^1^H NMR (CDCl_3_, δ ppm, *J*/Hz): 0.72 (s, 3H, 10-CH_3_); 0.85 (s, 3H, 8-CH_3_); 0.93 (s, 3H, 9-CH_3_); 1.29−1.49 (m, 2H, 5-CH_2_, 6-CH_2_); 1.60−1.68 (m, 2H, 3-CH_2_, 6-CH_2_); 1.79−1.98 (m, 2H, 5-CH_2_, 4-CH); 2.19−2.31 (m, 1H, 3-CH_2_); 2.44 (s, 3H, N-COCH_3_); 3.25−3.42 (m, 2H, 18-CH_2_, 2-CH); 3.76−4.03 (m, 10H, 18-CH_2_, C(22)-OCH_3_, C(23)-OCH_3_, C(24)-OCH_3_); 4.57 (d, *J* = 4.7 Hz, 4H, 1′-CH_2_, 1′′-CH_2_); 5.28−5.54 (m, 5H, 3′-CH_2_(H*cis*), 3′′-CH_2_(H*cis*), 19-CH, 3′-CH_2_(H*trans*), 3′′-CH_2_(H*trans*)); 6.04−6.13 (m, 2H, 2′-CH, 2′′-CH); 6.47 (m, 3H, 13-CH, 21-CH, 25-CH); 7.88 (d, *J* = 16.6 Hz, 1H, 16-CH). ^13^C NMR (CDCl_3_, δ ppm): 12.3 (10-CH_3_); 20 (8-CH_3_); 21.4 (9-CH_3_); 21.9 (N-COCH_3_); 27.5 (5-CH_2_); 34.2 (3-CH_2_); 39.7 (6-CH_2_); 44.4 (2-CH); 45.6 (4-CH); 45.8 (18-CH_2_); 48.1 (7-C); 49.6 (1-C); 56.1 (C(23)-OCH_3_); 60.1 (19-CH-N); 60.7 (C(22)-OCH_3_, C(24)-OCH_3_); 68.8 (1′-CH_2_); 69.9 (1′′-CH_2_); 97.2 (13-CH); 102.6 (25-CH); 102.7 (21-CH); 111.2 (11-C); 117.4 (3′-CH_2_); 118.1 (3′′-CH_2_); 125.4 (15-C); 128.5 (16-CH); 132.9 (2′-CH, 2′′-CH); 138.1 (20-C); 153.5 (22-C, 23-C, 24-C); 154.5 (17-C=N); 160.3 and 168.9 (12-C) and (14-C); 202.3 (C=O). ESI-MS *m*/*z*: found 603.81, [M + H]^+^, calcd. for C_36_H_47_N_2_O_6_ 603.76.

### 3.2. Antioxidant Activity

The antioxidant activity of pyrazoline derivatives was evaluated in vitro as inhibition of accumulation of secondary lipid peroxidation (LPO) products in substrates obtained from mouse brain homogenates (oil–water emulsion). The brain was homogenized in physiological saline (pH 7.4) (10% *v*/*v*) and centrifuged at 3000 rpm for 10 min. The low-speed supernatant was separated. The test compounds were added to the supernatant at final concentrations of 0.1 mM; then after 30 min, LPO was initiated by adding a freshly prepared solution of FeSO_4_ and ascorbic acid. Resveratrol and quercetin were taken as the most suitable reference compounds. Incubation of substrate was carried out in thermostated Biosan ES-20 shaker for 1 h at 37 °C. The concentration of secondary LPO products reacting with TBA (TBA reactive substances, TBA-RS) was determined at λ 532 nm using the extinction coefficient of 1.56 × 10^5^ M^−1^·cm^−1^ [37,46,47]. Absorption was measured using a Thermo Spectronic Genesys 20 instrument. Each experiment was repeated 4–8 times. Statistical analysis was assessed by applying Microsoft Office Excel 2010 software packages. Experimental data are presented as arithmetic mean values with indication of standard error of mean (SEM).

The assays were performed using the equipment of the Centre of Collective Usage Molecular Biology, Institute of Biology, Komi Scientific Centre, Ural Branch of the RAS.

## 4. Conclusions

In this work, derivatives of 3,5-diarylpyrazoline with various substituents (MeO, Hal, NO_2_, N(Me)_2_) were synthesized by the reaction of isobornylchalcones and hydrazine hydrate. All compounds were evaluated using an in vitro model of Fe^2+^/ascorbate-initiated lipid peroxidation in a substrate containing laboratory mouse brain lipids. According to in vitro studies, pyrazoline **7i** containing a hydroxyl group in the 2′-position in ring A and a catechol fragment in ring B was the most active antioxidant.

## Figures and Tables

**Table 1:** Yields of pyrazolines.

Compound	R	Yield, %	Compound	R	Yield, %
**7a**	3-NO_2_	78	9a	3-NO_2_	85
**7b**	4-Cl	70	9b	4-Cl	76
**7c**	4-Br	91	9c	4-Br	91
**7d**	4-NMe_2_	71	9d	4-NMe_2_	68
**7e**	2-OMe	96	9e	2-OMe	93
**7f**	3-OMe	91	9f	3-OMe	77
**7g**	4-OMe	90	9g	4-OMe	82
**7h**	2,3-OMe	90	9h	2,3-OMe	92
**7i**	3,4-OMe	99	9i	3,4-OMe	99
**7j**	2,4,6-OMe	75	9j	2,4,6-OMe	56
**7k**	3,4,5-OMe	98	9k	3,4,5-OMe	96

## Data Availability

Not applicable.

## Supplementary Materials

The following are available online. Figure S1: ^1^H NMR (CDCl_3_) spectrum of compound **7a**. Figure S2: ^13^C NMR (CDCl_3_) spectrum of compound **7a**. Figure S3: ^1^H NMR (CDCl_3_) spectrum of compound **7b**. Figure S4: ^13^C NMR (CDCl_3_) spectrum of compound **7b**. Figure S5: ^1^H NMR (CDCl_3_) spectrum of compound **7i**. Figure S6: ^13^C NMR (CDCl_3_) spectrum of compound **7i**. Figure S7: ^1^H NMR (CDCl_3_) spectrum of compound **7j**. Figure S8: ^13^C NMR (CDCl_3_) spectrum of compound **7j**. Figure S9: ^1^H NMR (CDCl_3_) spectrum of compound **9a**. Figure S10: ^13^C NMR (CDCl_3_) spectrum of compound **9a**. Figure S11: ^1^H NMR (CDCl_3_) spectrum of compound **9b**. Figure S12: ^13^C NMR (CDCl_3_) spectrum of compound **9b**. Figure S13: ^1^H NMR (CDCl_3_) spectrum of compound **9i**. Figure S14: ^13^C NMR (CDCl_3_) spectrum of compound **9i**. Figure S15: ^1^H NMR (CDCl_3_) spectrum of compound **9k**. Figure S16: ^13^C NMR (CDCl_3_) spectrum of compound **9k**.

## Sample Availability

Samples of the compounds **6a**–**k**, **7a**–**k**, **8a**–**k**, **9a**–**k** are available from the authors.
