# Supplementary material for: Isobornylchalcones as Scaffold for the Synthesis of Diarylpyrazolines with Antioxidant Activity
Source: Molecules. 2021 Jun 11;26(12):3579. doi: 10.3390/molecules26123579 (PMC8230786; doi:10.3390/molecules26123579)
Supplement: Supplementary file 1 [file molecules-26-03579-s001.zip › molecules-1255456-supplementary.pdf]

Supplementary Material

Isobornylchalcones as scaffold for the synthesis of diarylpyrazolines with antioxidant activity

Svetlana A. Popova [1,*], Evgenia V. Pavlova [1], Oksana G. Shevchenko [2], Irina Yu. Chukicheva [1] and Aleksandr V. Kutchin [1]

[1] Institute of Chemistry, Komi Scientific Centre, Ural Branch of the Russian Academy of Sciences, 48, Pervomayskaya, 167000, Syktyvkar, Russian Federation; chukicheva-iy@chemi.komisc.ru; evgenia.pavlova92@rambler.ru; kutchin-av@mail.ru

[2] Institute of Biology, Komi Scientific Centre, Ural Branch of the Russian Academy of Sciences, 28, Kommunisticheskaya, 167982, Syktyvkar, Russian Federation; shevchenko@ib.komisc.ru

* Correspondence: popova-sa@chemi.komisc.ru; Tel.: +7-8212-21 84 77

Table of Contents

^{1}H and ^{13}C NMR spectra of compounds **7a**, **7b**, **7i**, **7j**, **9a**, **9b**, **9i**, **9k** S2

1-(5-(3-Nitrophenyl)-3-(4'-allyloxy-2'-hydroxy-5'-isobornylphenyl)-4,5-dihydro-(1H)-pyrazole-1-yl)etanone (7a)

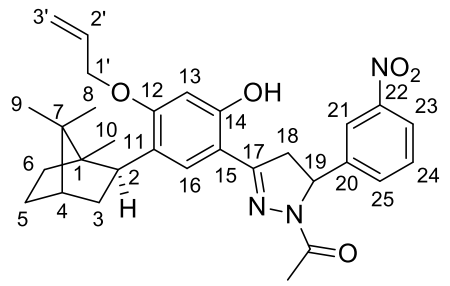

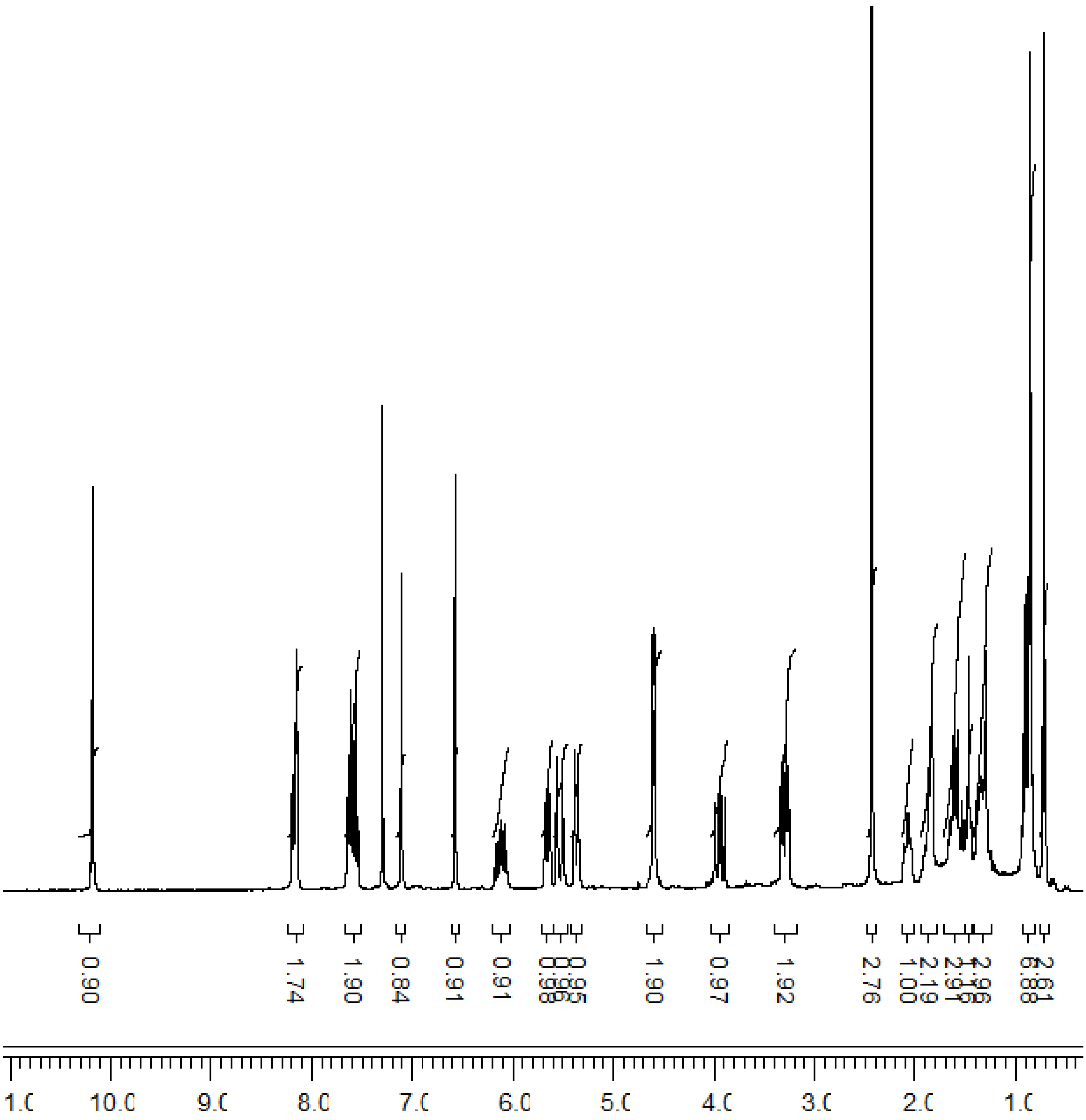

Figure S1: ^{1}H NMR ($CDCl_3$) spectrum of compound **7a**.

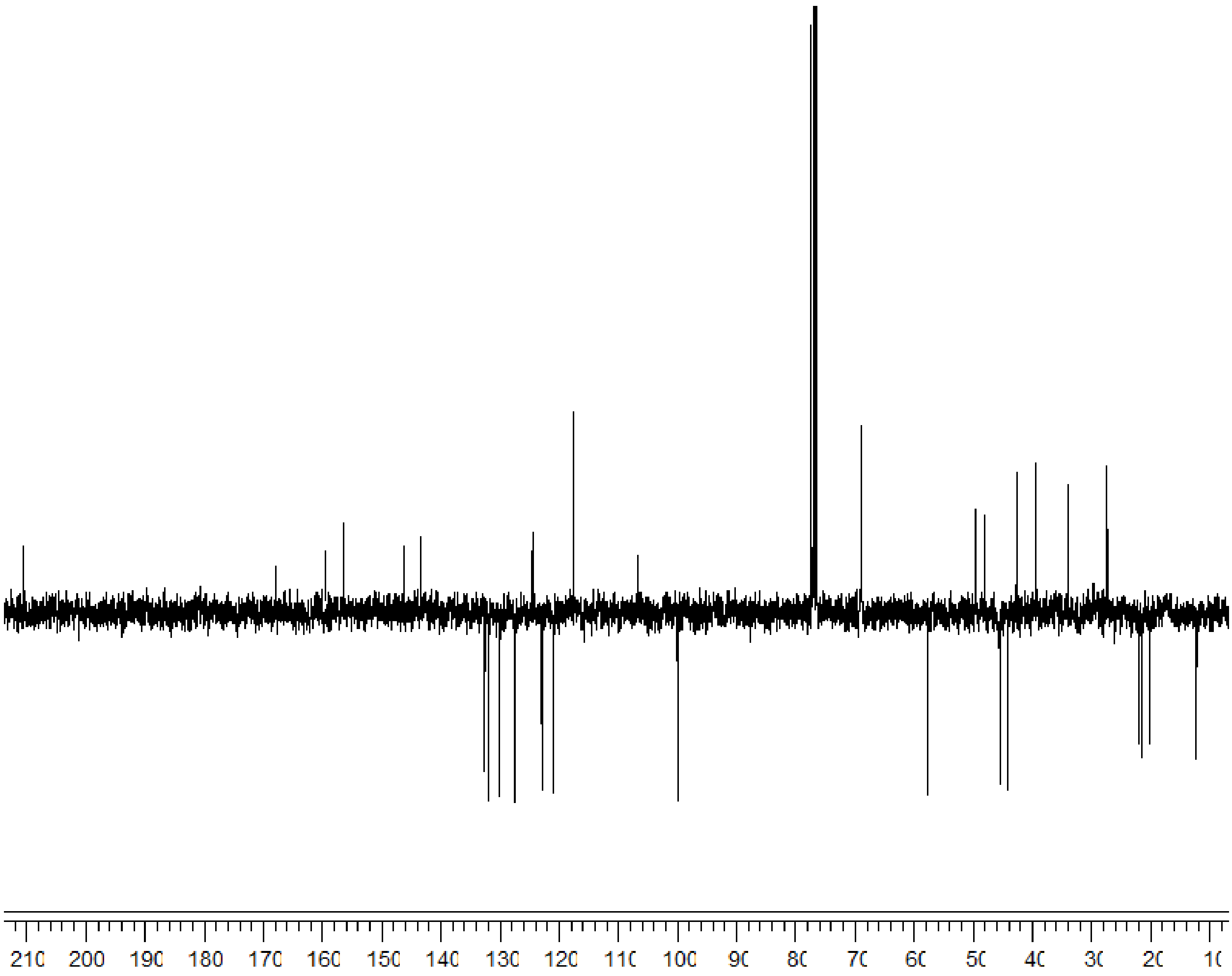

Figure S2: ^{13}C NMR ($CDCl_3$) spectrum of compound **7a**.

1-(5-(4-Chlorophenyl)-3-(4'-allyloxy-2'-hydroxy-5'-isobornylphenyl)-4,5-dihydro-(1H)-pyrazole-1-yl)etanone (7b)

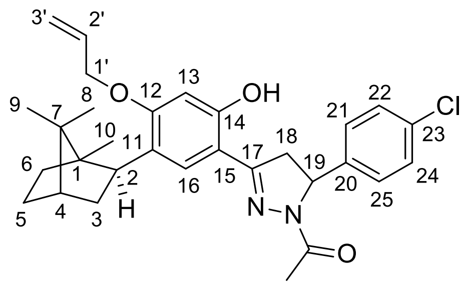

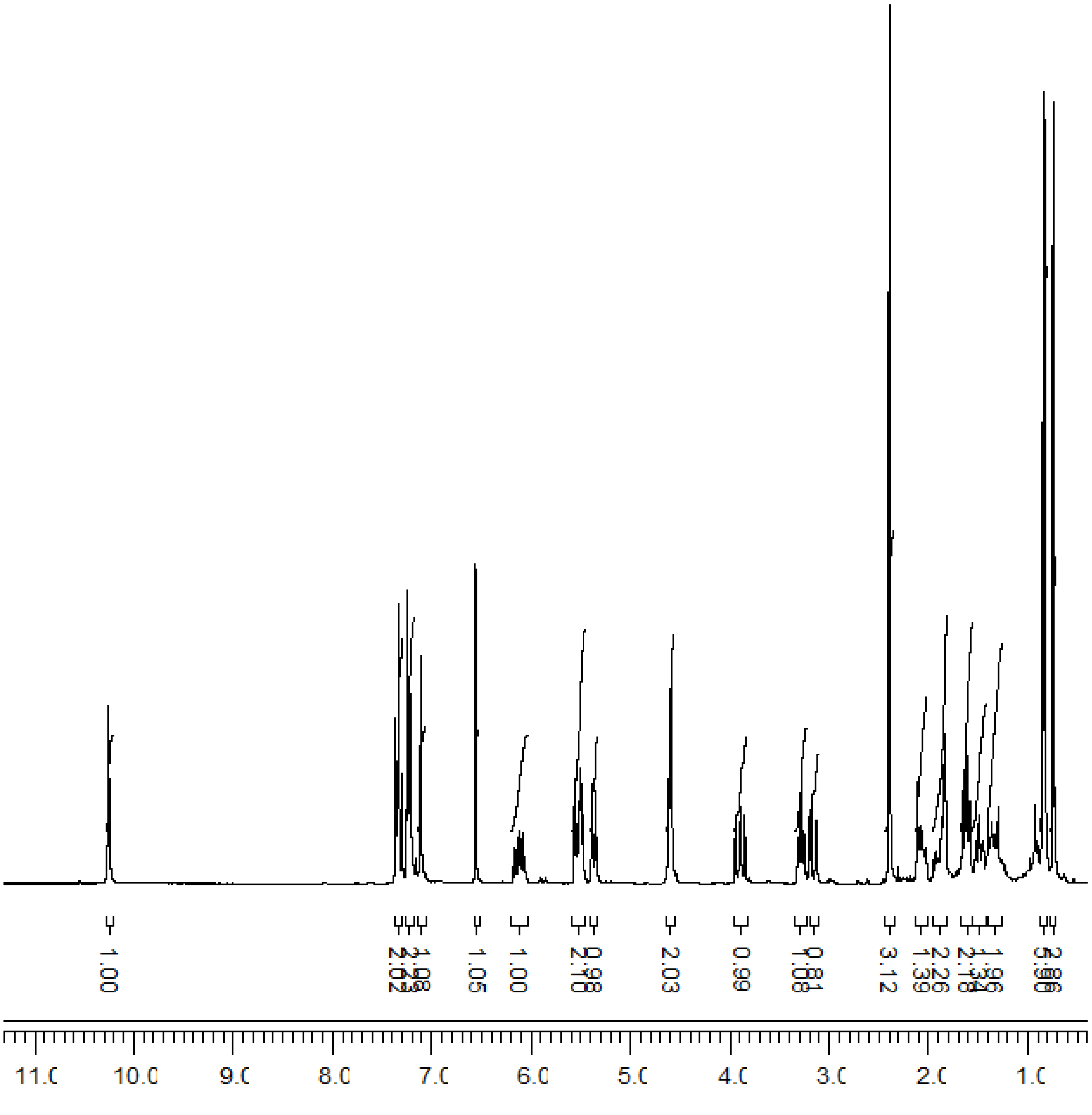

Figure S3: ^{1}H NMR ($CDCl_3$) spectrum of compound **7b**.

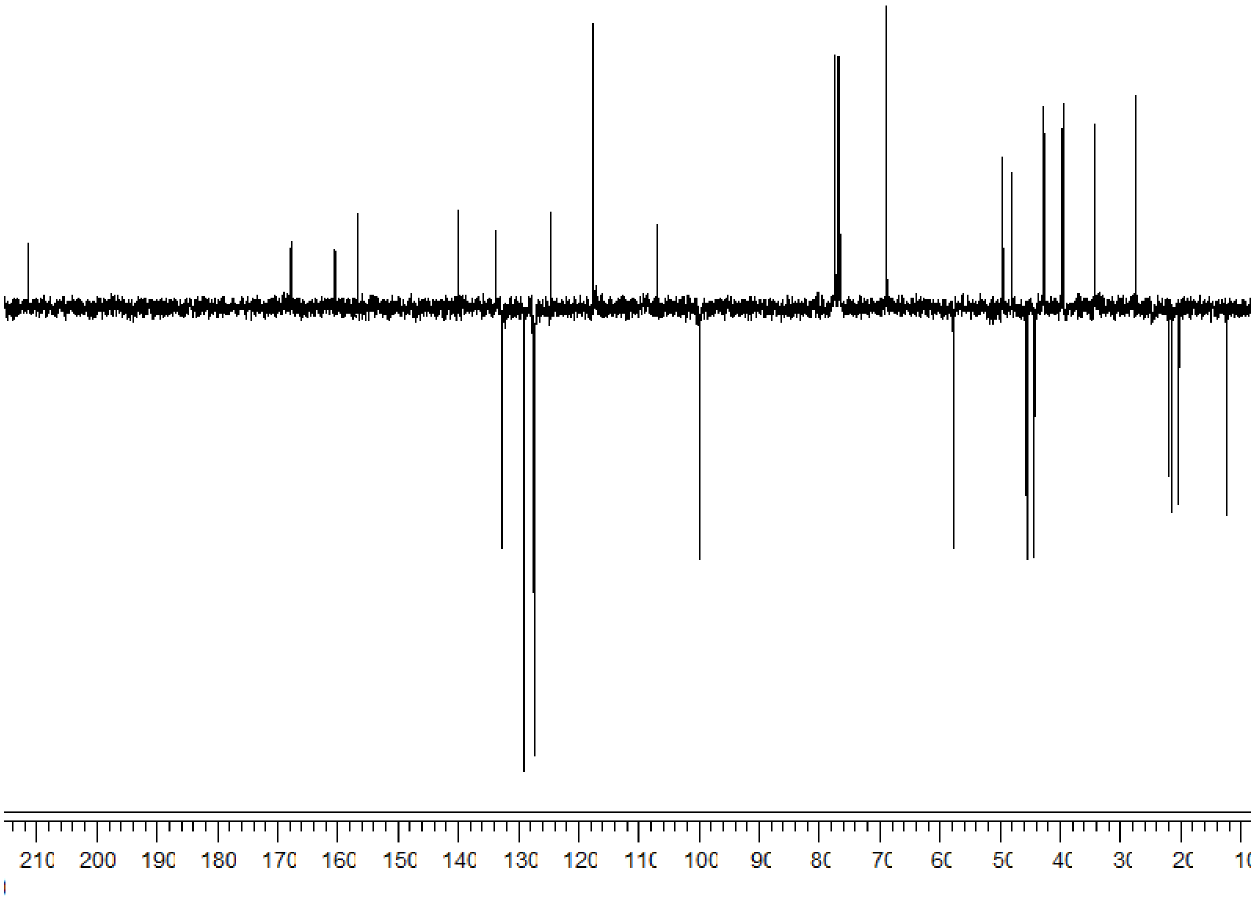

Figure S4: ^{13}C NMR ($CDCl_3$) spectrum of compound **7b**.

1-(5-(3,4-Dimethoxyphenyl)-3-(4'-allyloxy-2'-hydroxy-5'-isobornylphenyl)-4,5-dihydro-(1H)-pyrazole-1-yl)etanone (7i)

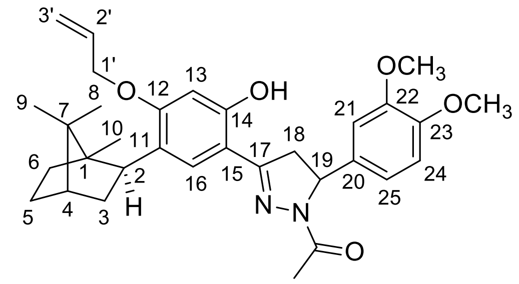

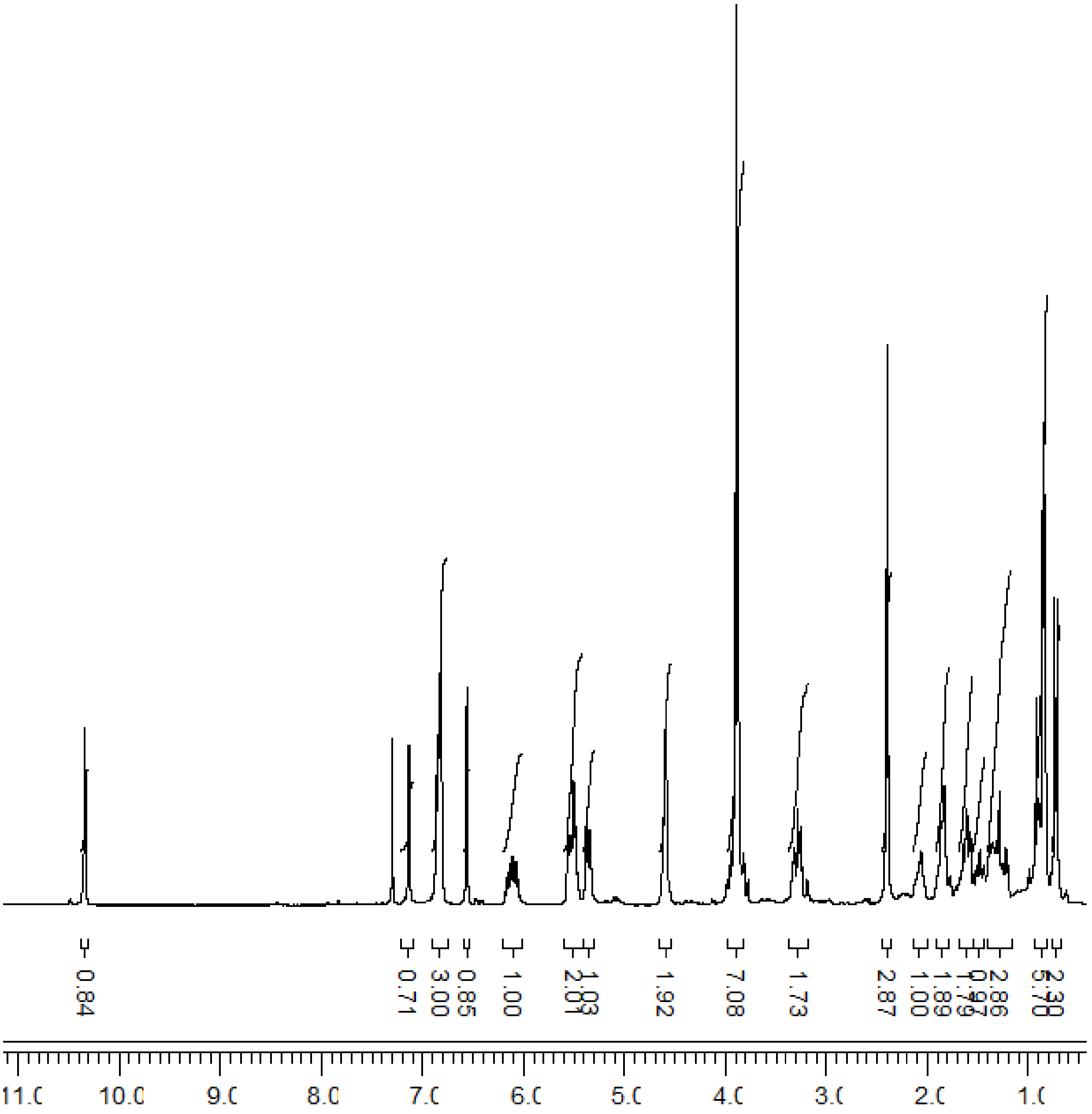

Figure S5: ^{1}H NMR ($CDCl_3$) spectrum of compound **7i**.

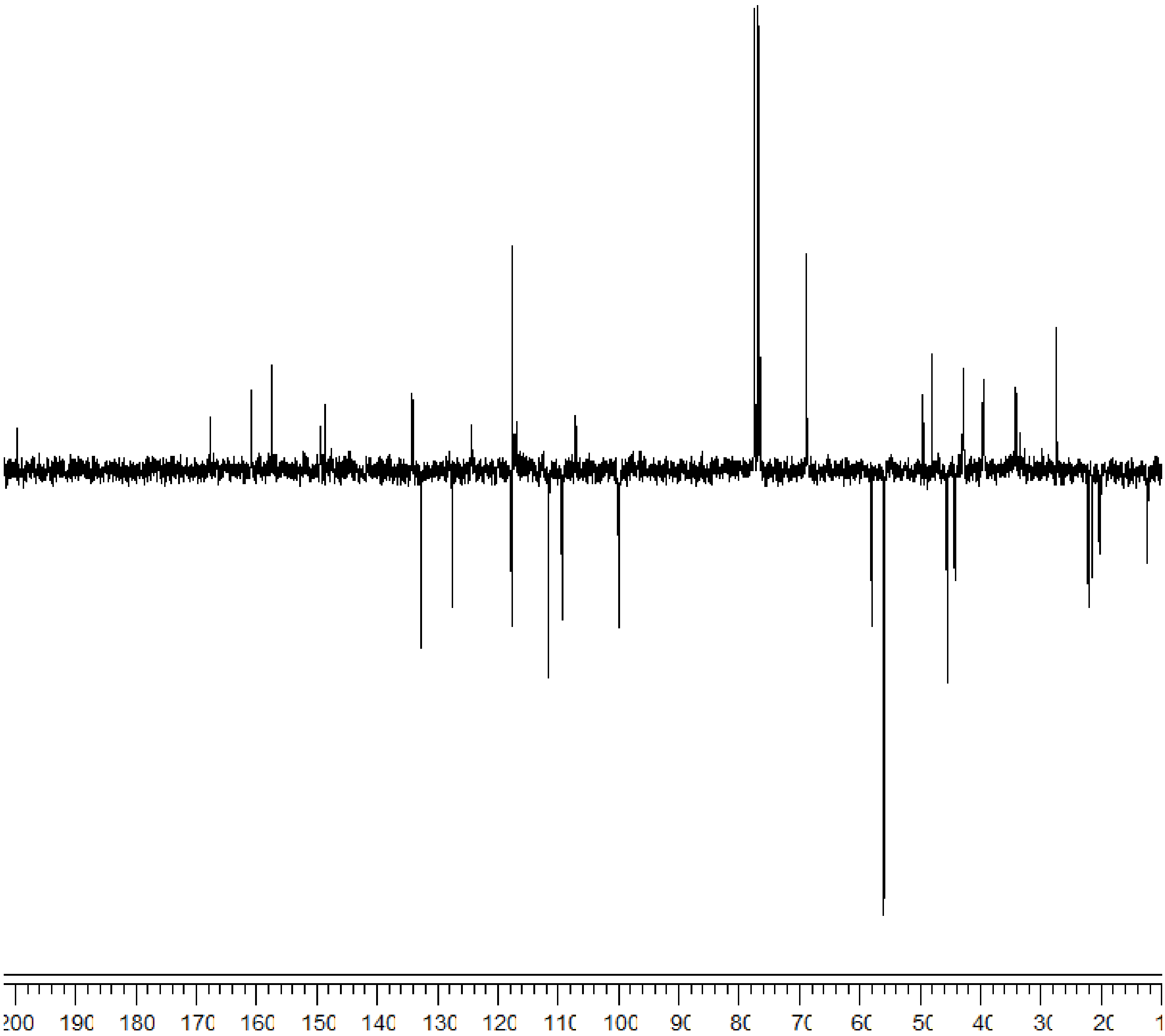

Figure S6: ^{13}C NMR ($CDCl_3$) spectrum of compound **7i**.

1-(5-(2,4,6-Trimethoxyphenyl)-3-(4'-allyloxy-2'-hydroxy-5'-isobornylphenyl)-4,5-dihydro-(1H)-pyrazole-1-yl)etanone (7j)

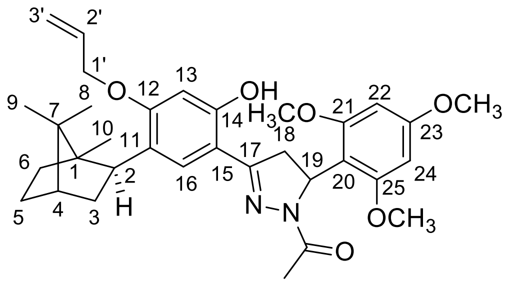

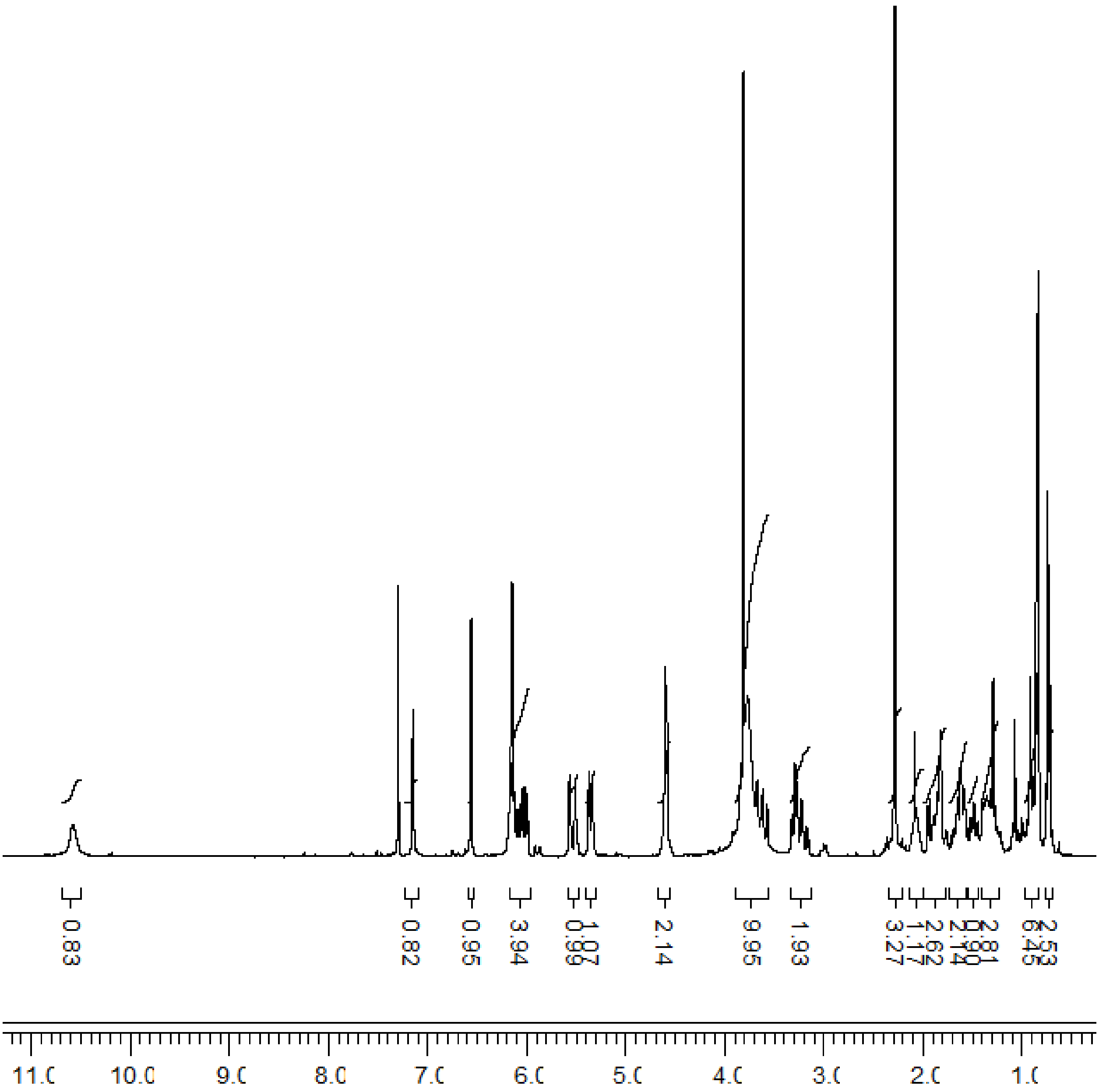

Figure S7: ^{1}H NMR ($CDCl_3$) spectrum of compound **7j**.

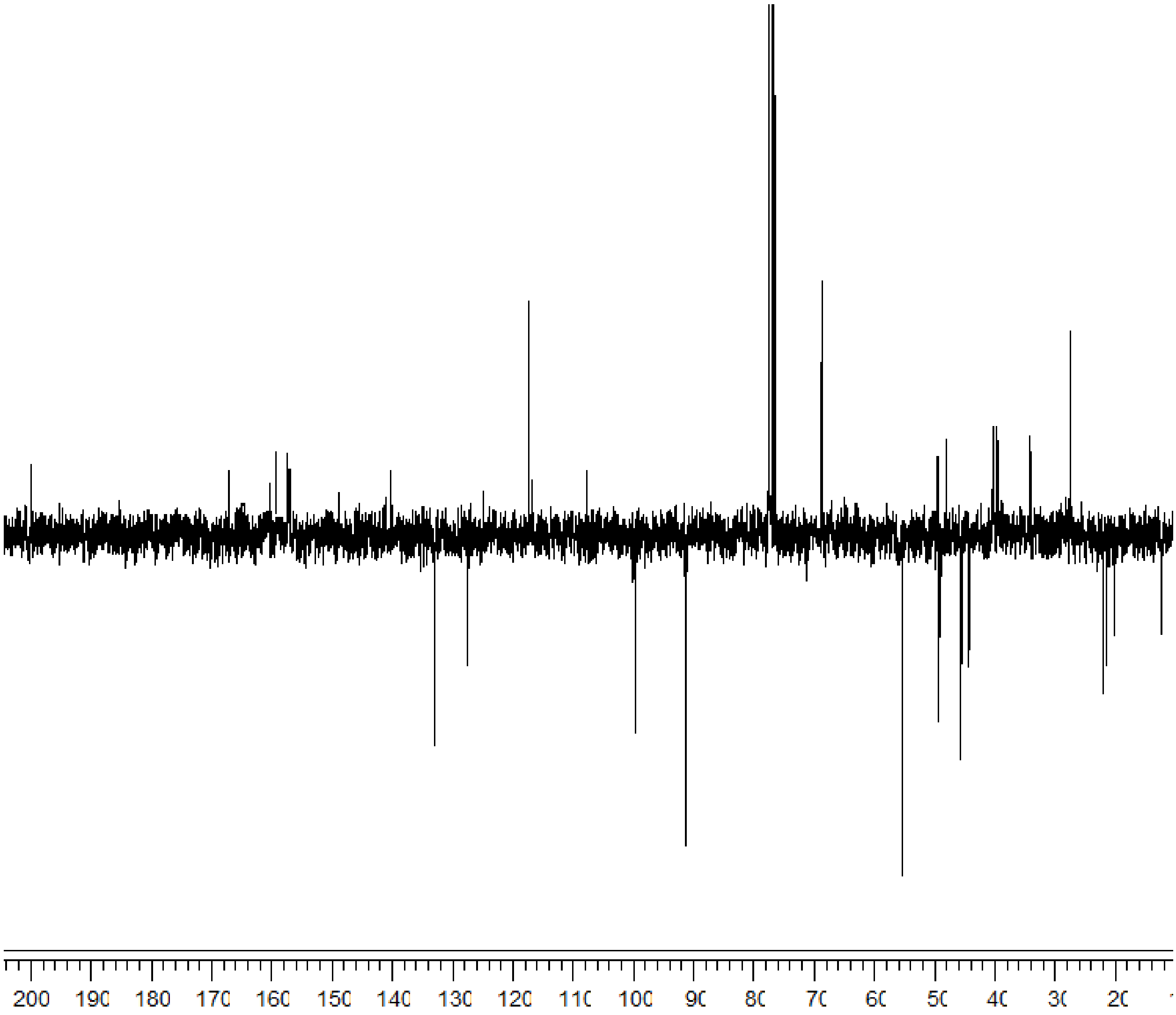

Figure S8: ^{13}C NMR ($CDCl_3$) spectrum of compound **7j**.

1-(5-(3-Nitrophenyl)-3-(2',4'-diallyloxy-5'-isobornylphenyl)-4,5-dihydro-(1H)-pyrazole-1-yl)etanone (9a)

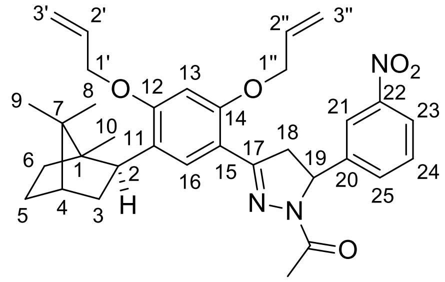

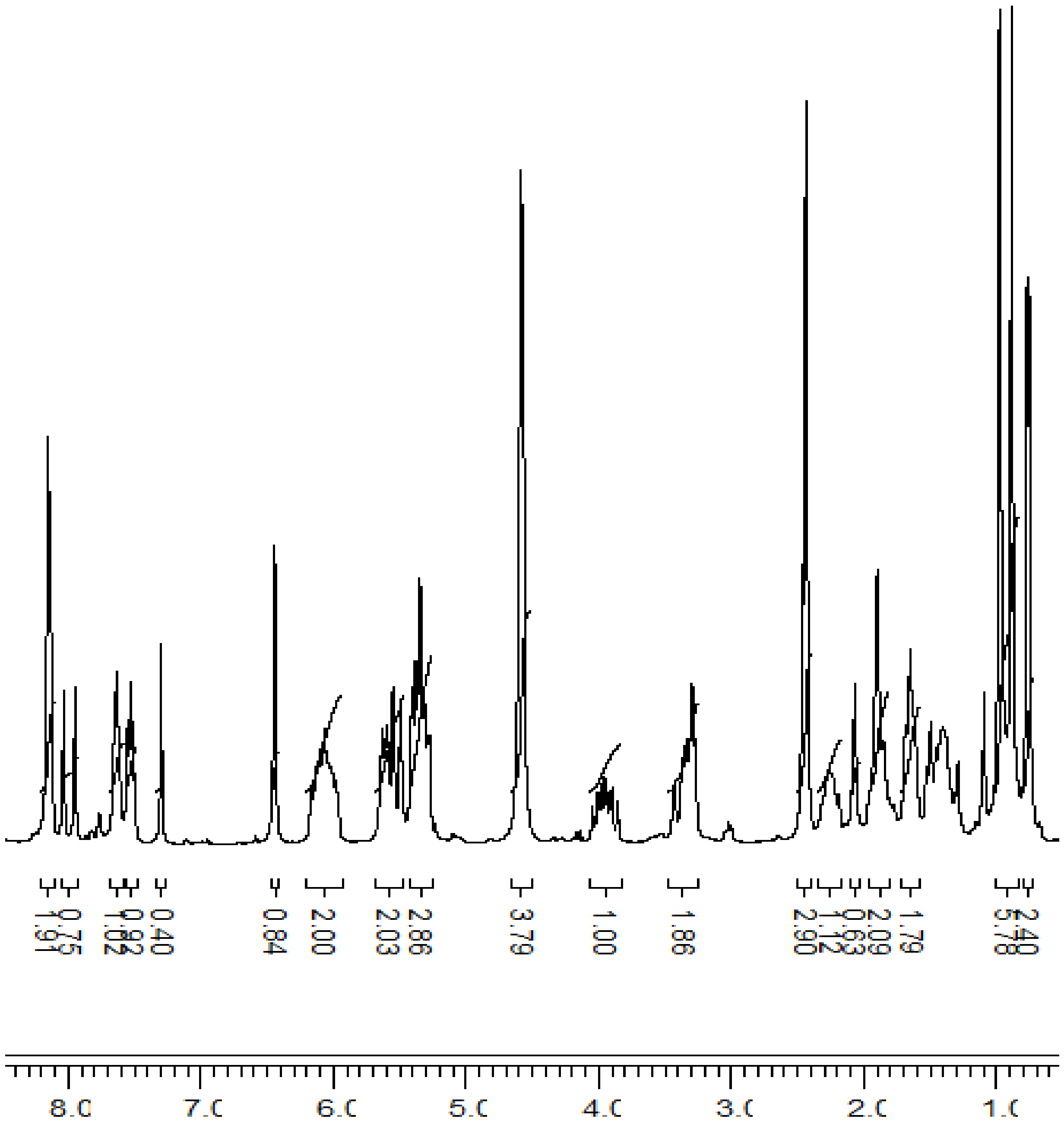

Figure S9: ^{1}H NMR (CDCl$_3$) spectrum of compound **9a**.

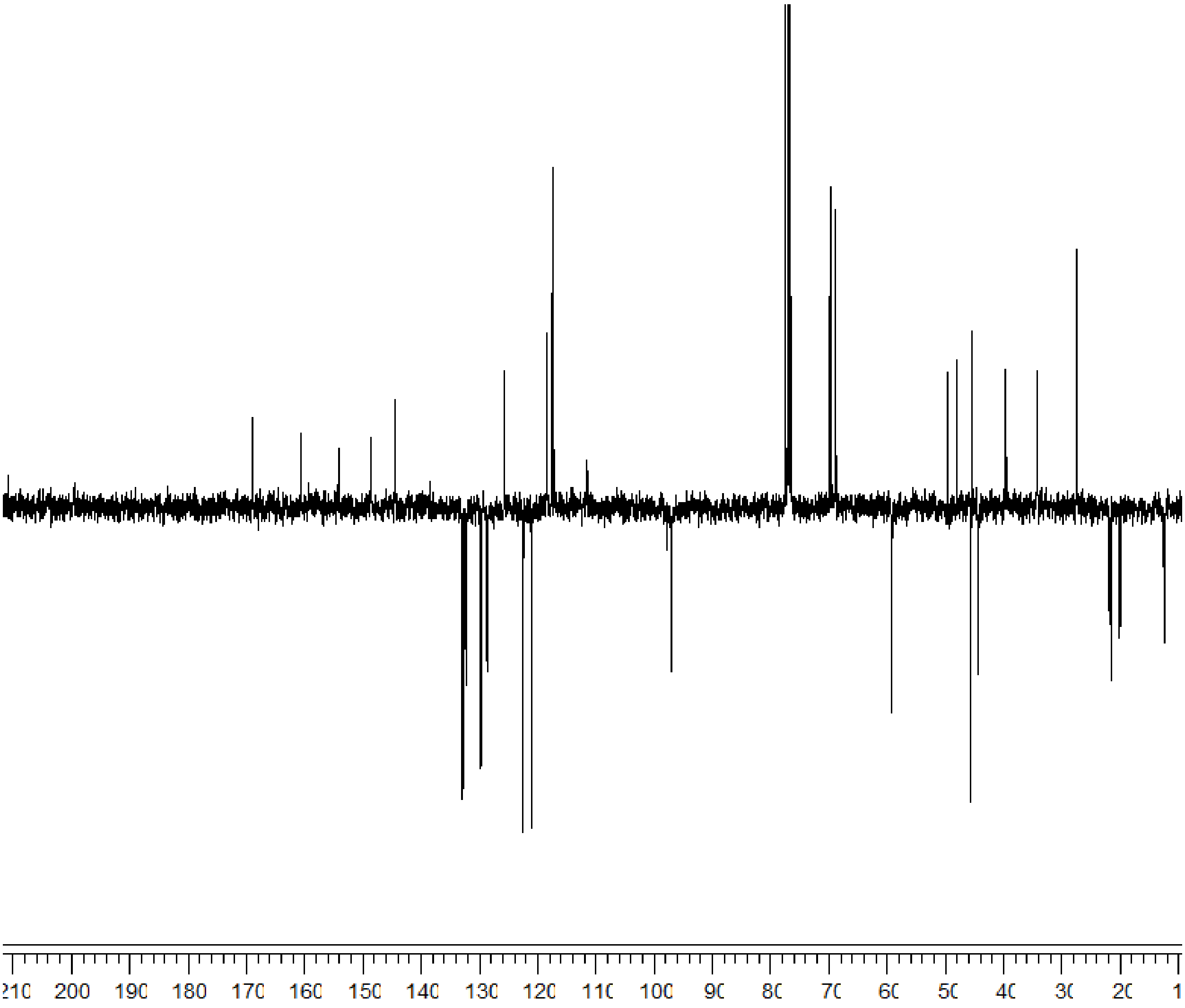

Figure S10: ^{13}C NMR ($CDCl_3$) spectrum of compound **9a**.

1-(5-(4-Clorophenyl)-3-(2',4'-diallyloxy-5'-isobornylphenyl)-4,5-dihydro-(1H)-pyrazole-1-yl)etanone (9b)

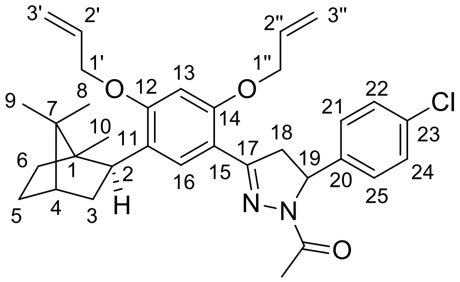

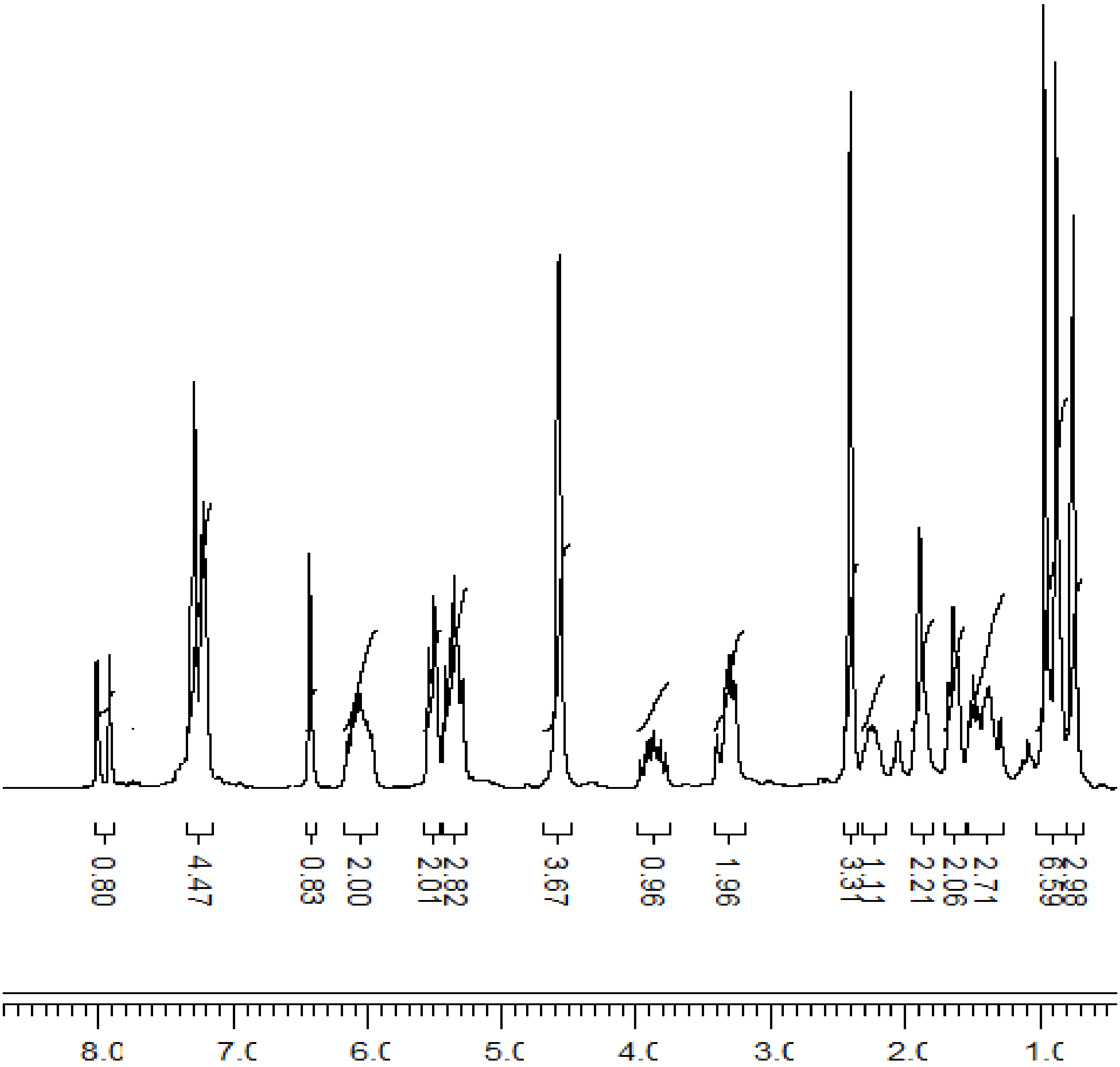

Figure S11: ^{1}H NMR ($CDCl_3$) spectrum of compound **9b**.

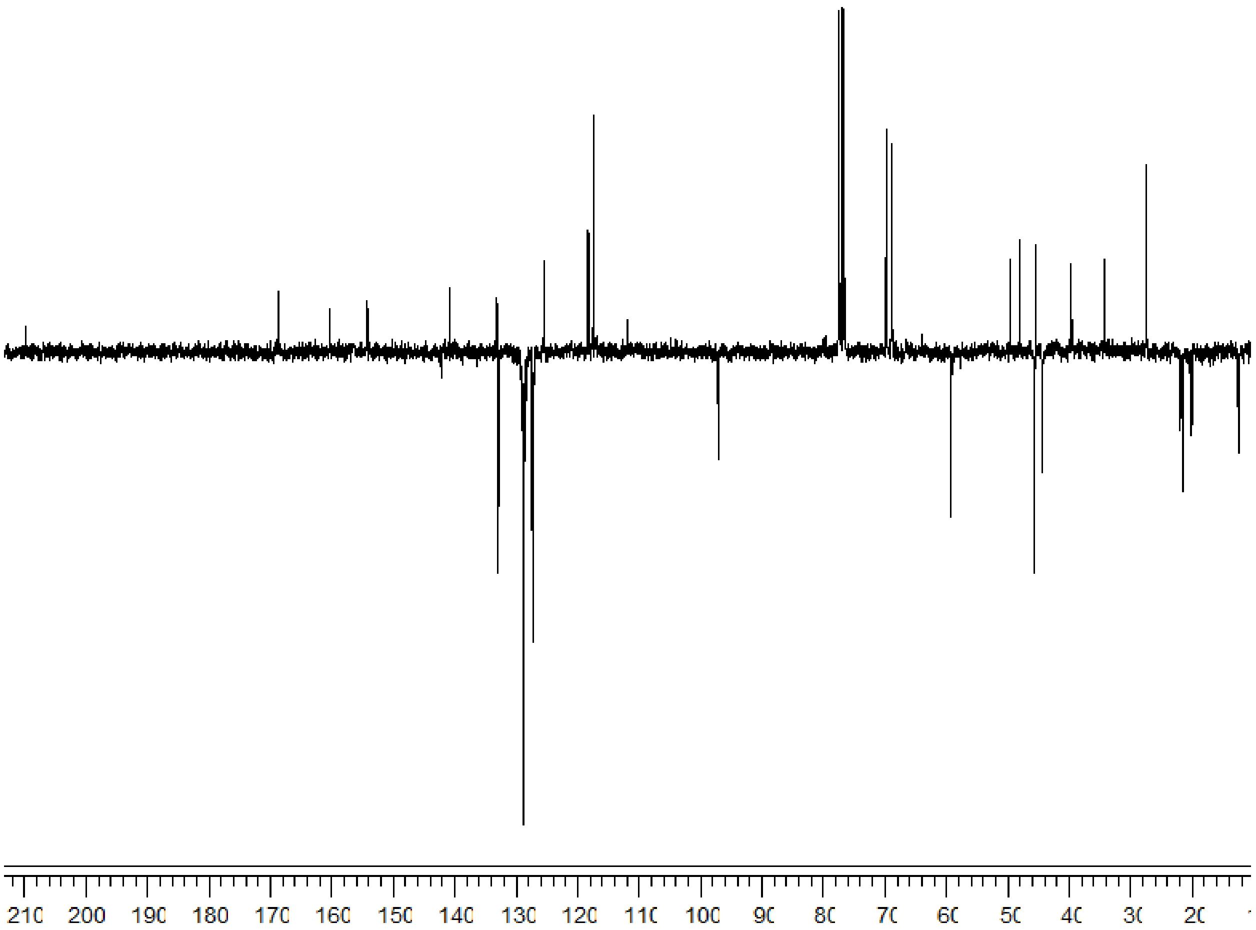

Figure S12: ^{13}C NMR ($CDCl_3$) spectrum of compound **9b**.

1-(5-(3,4-Dimethoxyhenyl)-3-(2',4'-diallyloxy-5'-isobornylphenyl)-4,5-dihydro-(1H)-pyrazole-1-yl)etanone (9i)

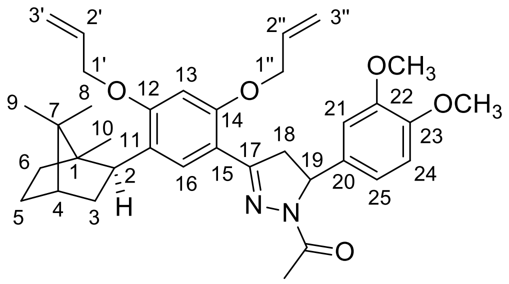

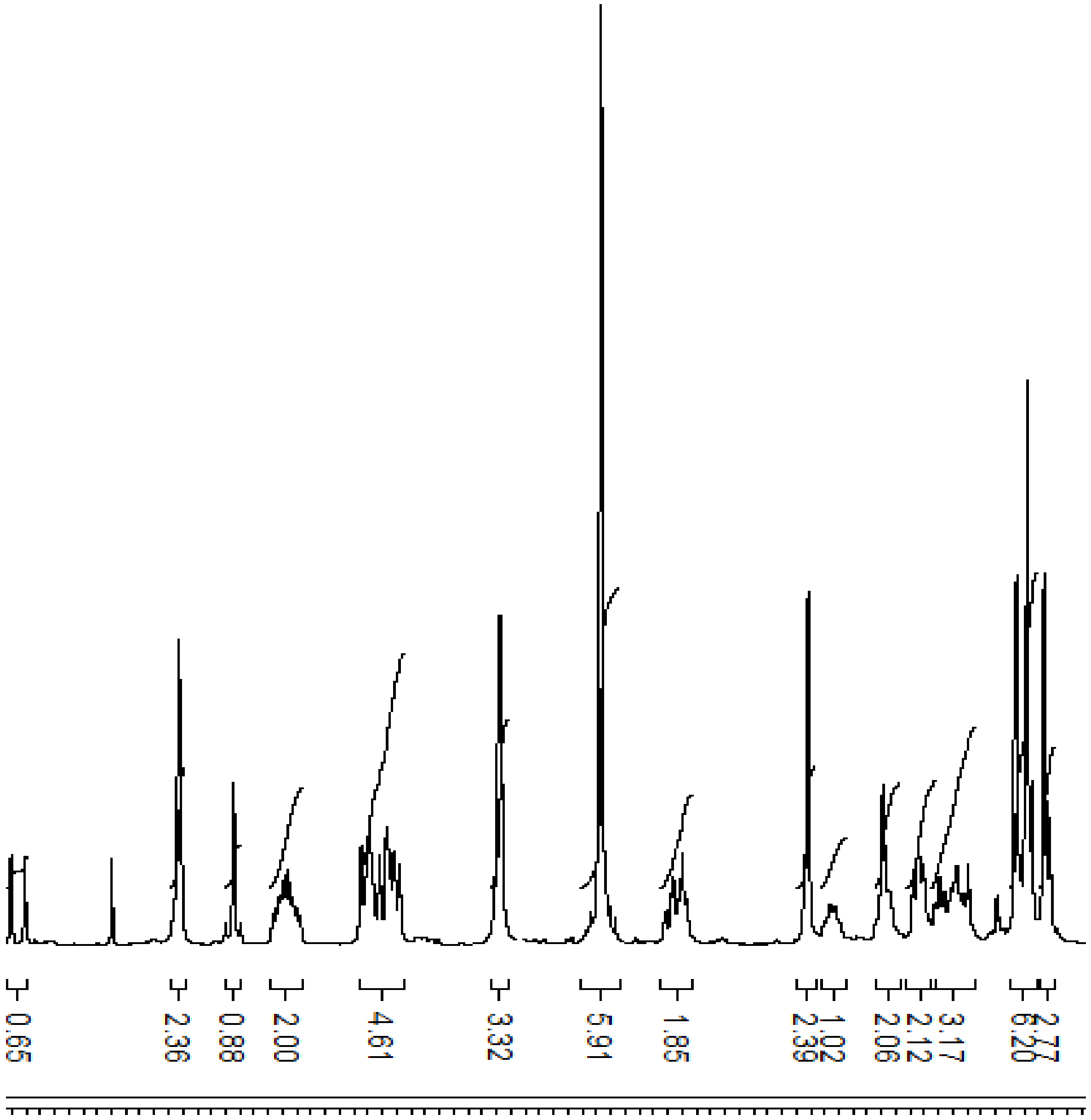

Figure S13: ^{1}H NMR ($CDCl_3$) spectrum of compound **9i**.

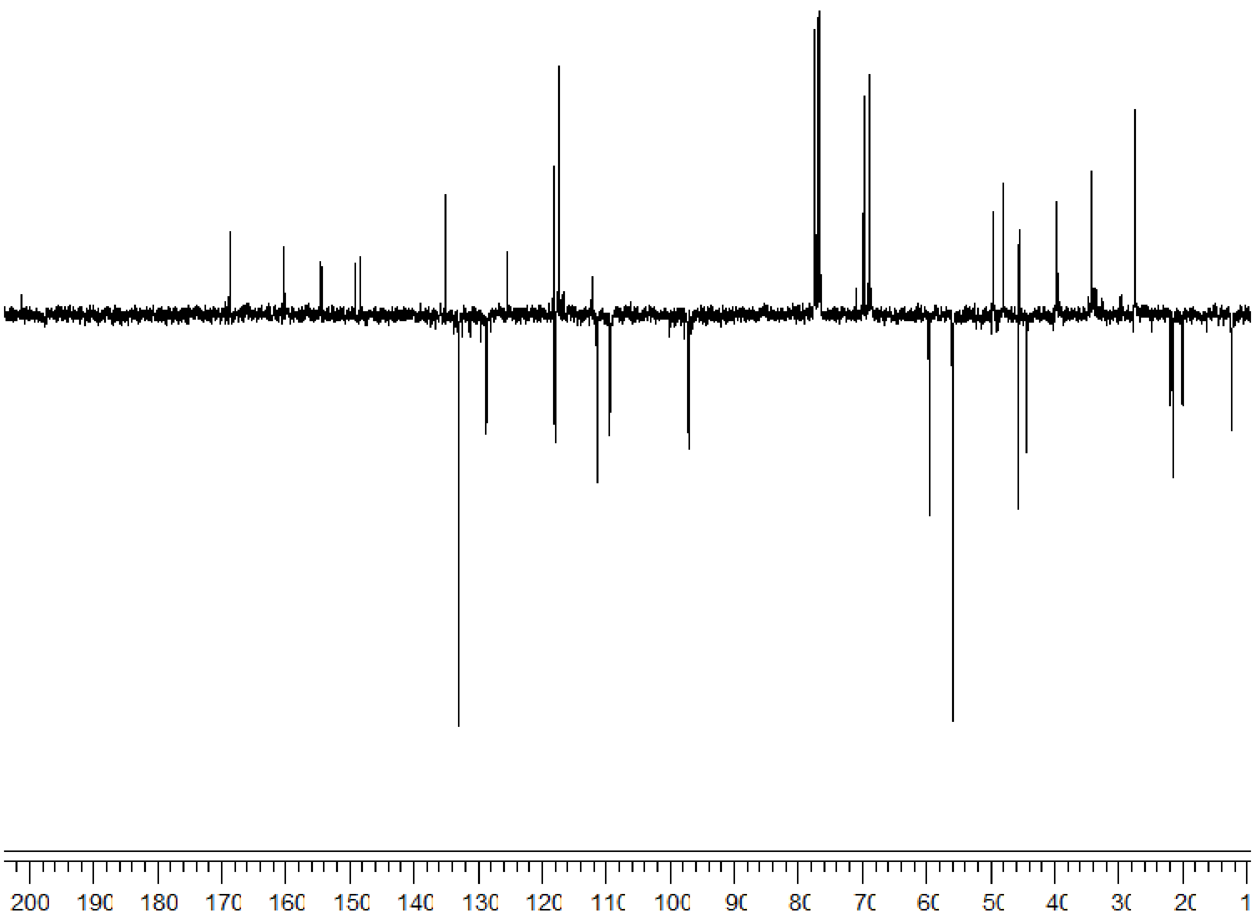

Figure S14: ^{13}C NMR ($CDCl_3$) spectrum of compound **9i**.

1-(5-(3,4,5-Trimethoxyhenyl)-3-(2',4'-diallyloxy-5'-isobornylphenyl)-4,5-dihydro-(1H)-pyrazole-1-yl)etanone (9k)

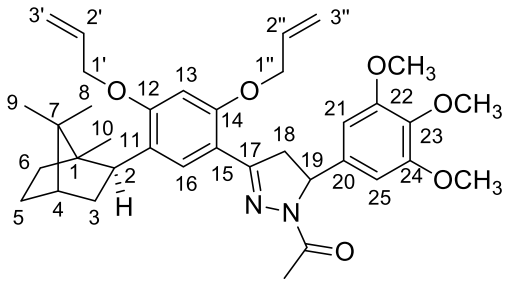

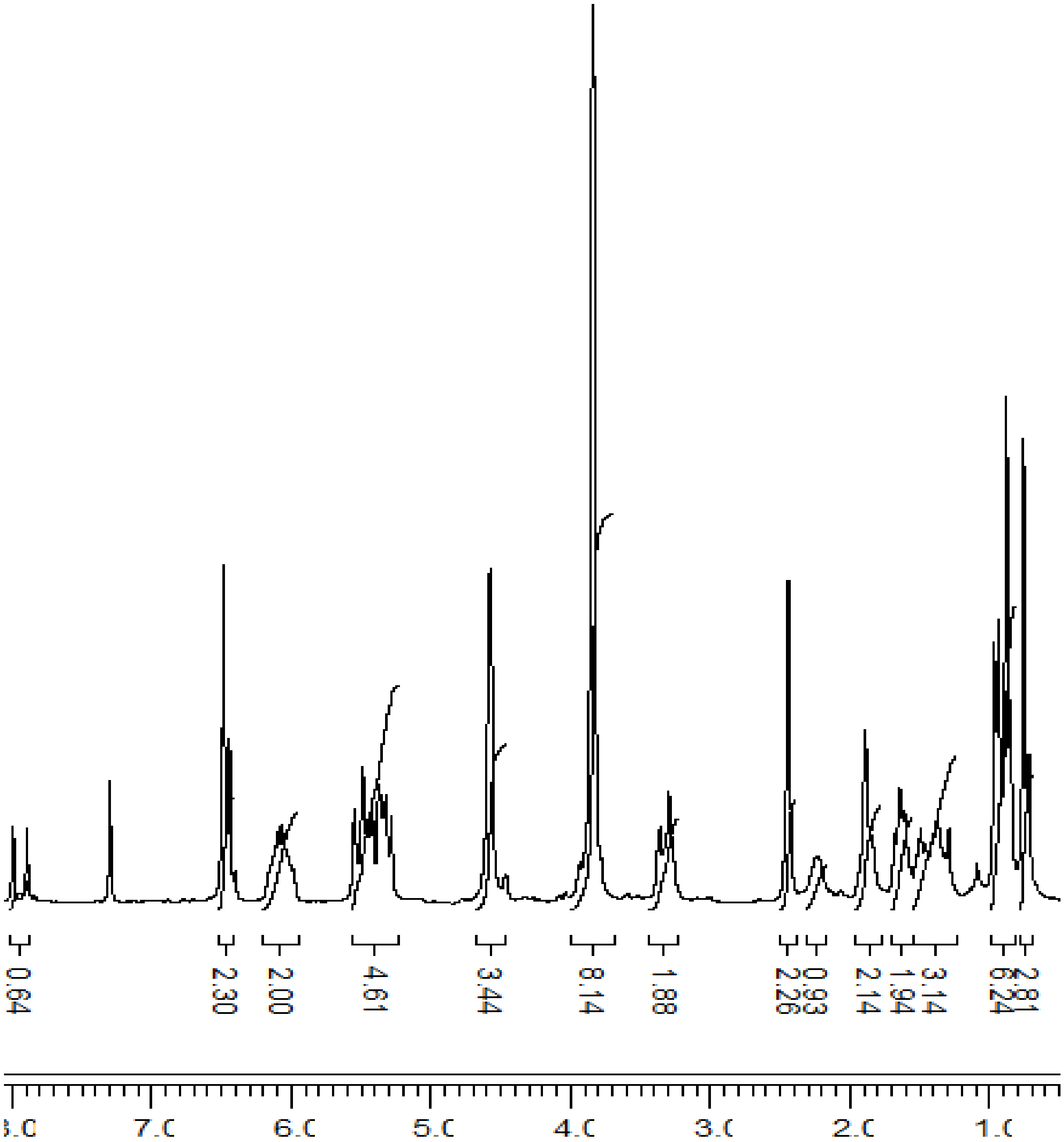

Figure S15: ^{1}H NMR ($CDCl_3$) spectrum of compound **9k**.

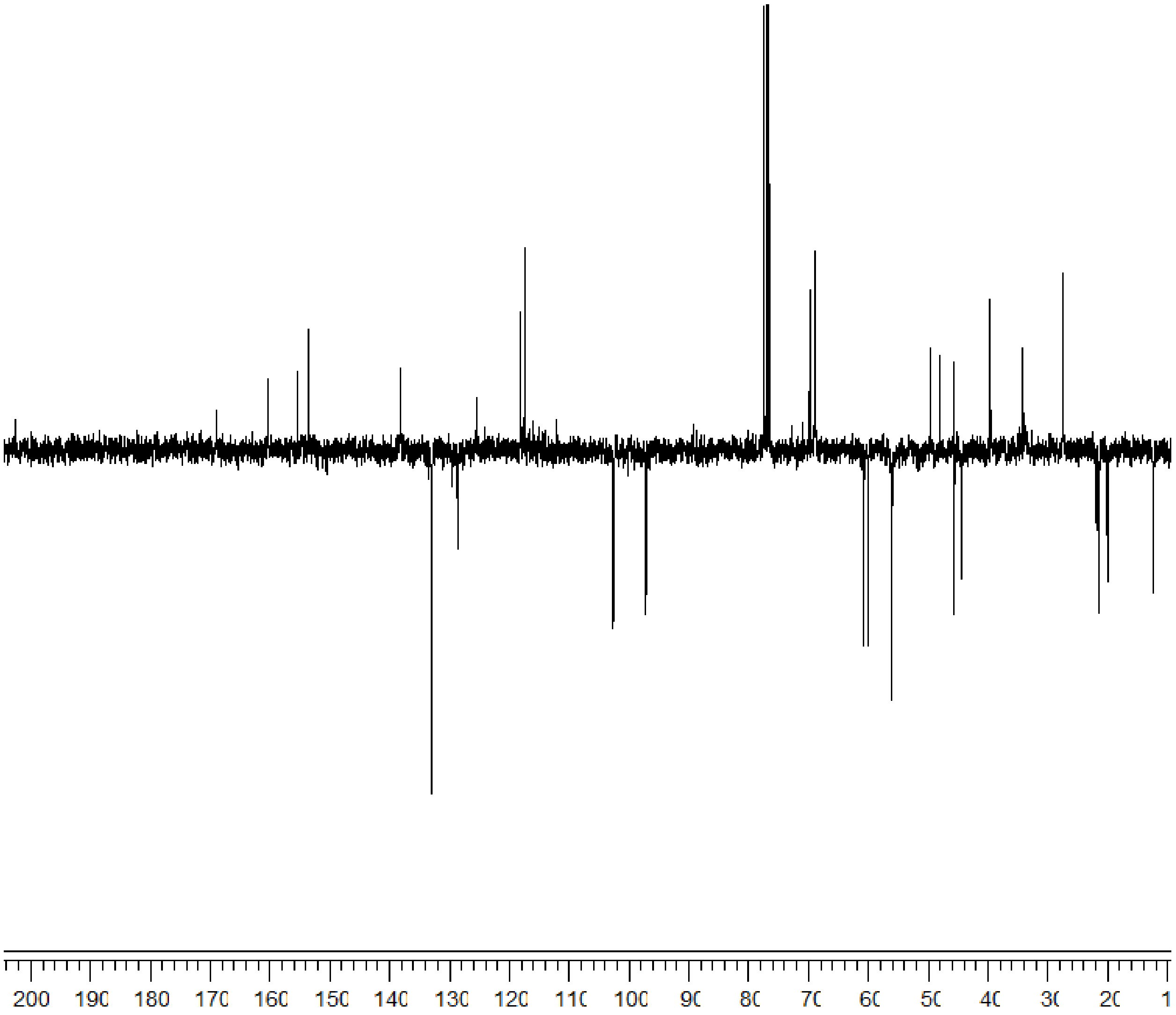

Figure S16: ^{13}C NMR ($CDCl_3$) spectrum of compound **9k**.
